# Ocular adverse events associated with antibody-drug conjugates in oncology: a pharmacovigilance study based on FDA adverse event reporting system (FAERS)

**DOI:** 10.3389/fphar.2024.1425617

**Published:** 2024-08-20

**Authors:** KaiLi Mao, Ping Chen, HuaYu Sun, SongYang Zhong, HongLiang Zheng, LuYao Xu

**Affiliations:** Department of Pharmacy, The Quzhou Affiliated Hospital of Wenzhou Medical University, Quzhou People’s Hospital, Quzhou, Zhejiang, China

**Keywords:** antibody-drug conjugates (ADCs), ocular toxicity, FAERS, pharmacovigilance, data mining

## Abstract

**Background:**

Antibody-drug conjugates (ADCs) have emerged as the focus and hotspots in the cancer field, yet the accompanying ocular toxicity has often been underestimated. We aimed to comprehensively and comparatively analyze the risk of ocular toxicity associated with various ADCs using the FDA Adverse Event Reporting System (FAERS) database.

**Methods:**

Data were extracted from the FAERS database from Q3 2011 to Q3 2023. We analyzed the clinical characteristics of ADCs-related ocular adverse events (AEs). These data were further mined by proportional analysis and Bayesian approach to detect signals of ADCs-induced ocular AEs. Moreover, the time to onset of ocular toxicity was also evaluated.

**Results:**

A total of 1,246 cases of ocular AEs were attributed to ADCs. Ocular toxicity signals were observed in patients treated with belantamab mafodotin, brentuximab vedotin, enfortumab vedotin, mirvetuximab soravtansine, sacituzumab govitecan, trastuzumab deruxtecan, and trastuzumab emtansine. Of these, belantamab mafodotin, trastuzumab emtansine, and mirvetuximab soravtansine, whose payloads are microtubule polymerization inhibitors, were more susceptible to ocular toxicity. The ten most common ADCs-related ocular AEs signals are keratopathy [ROR = 1,273.52, 95% CI (1,129.26–1,436.21)], visual acuity reduced [ROR = 22.83, 95% CI (21.2–24.58)], dry eye [ROR = 9.69, 95% CI (8.81–10.66)], night blindness [ROR = 259.87, 95% CI (228.23–295.89)], vision blurred [ROR = 1.78, 95% CI (1.57–2.02)], photophobia [ROR = 10.45, 95% CI (9.07–12.05)], foreign body sensation in eyes [ROR = 23.35, 95% CI (19.88–27.42)], ocular toxicity [ROR = 144.62, 95% CI (117.3–178.32)], punctate keratitis [ROR = 126.21, 95% CI (101.66–156.69)], eye disorder [ROR = 2.71, 95% CI (2.21–3.32)]. In terms of onset time, sacituzumab govitecan displayed an earlier onset of 21 days, while trastuzumab deruxtecan exhibited the latest onset of 223 days.

**Conclusion:**

ADCs may increase the risk of ocular toxicity in cancer patients, leading to serious mortality. With the widespread application of newly launched ADCs, combining the FAERS data with other data sources is crucial for monitoring the ocular toxicity of ADCs. In addition, novel ocular toxicity signals not documented in product labeling were detected. Further research will be necessary to validate our findings in the future.

## 1 Introduction

Antibody-drug conjugates (ADCs) are a class of immunoconjugates consisting of highly selective monoclonal antibodies and cytotoxic drugs (also called payload) linked together by cleavable or non-cleavable chemical linkers, combining the potent killing effect of traditional small molecule chemotherapy with the tumor-targeting properties of antibody drugs (Sheikh and Huang, 2023). Novel potential anticancer drugs of antibody-drug conjugates (ADCs) have attracted much attention in recent years. ADCs are like precision-guided “biological missiles” that can precisely destroy cancer cells, increase the therapeutic window, and reduce off-target side effects (Criscitiello et al., 2021). The combination of these drugs can achieve specific targeting and efficient killing of cancer cells. Thus, ADCs have made significant progress in oncology in recent years and have been rapidly applied in the clinic (Teicher, 2014; Hayashi and Hinata, 2022).

The inception and clinical trials of ADCs began in the 1990s and were subsequently incorporated into many oncology clinics, and the pace of development continues to accelerate (Khongorzul et al., 2020). Fifteen ADCs have been approved by the Food and Drug Administration (FDA) for various treatments of hematologic and solid tumors, and hundreds more studies and clinical trials are currently underway. The primary role of monoclonal antibody moiety in ADCs is to target tumor cells, preferably by binding to antigens that are highly expressed in malignant cells but poorly or not expressed in normal cells. Once directed to the tumor cells by the monoclonal antibodies, the conjugate is internalized and undergoes lysosomal degradation, releasing a cytotoxic payload that acts on the intracellular target (Lambert and Morris, 2017; Hafeez et al., 2020). Until now, target antigens for which ADCs have been approved include HER2, Trop2, Nectin4, and EGFR in solid tumors and CD19, CD22, CD33, CD30, BCMA, and CD79b in hematological malignancies (Maiti et al., 2023). Another property of the antibody component lies in triggering the internalization of the complex, thus enabling the delivery of the payload. The payload is a key part of ADCs and has great diversity. Currently, FDA-approved ADCs with payloads include anti-mitotic agents [monomethyl auristatin E (MMAE), monomethyl auristatin F (MMAF), and medenosine derivatives (DM1 and DM4)], DNA-damaging agents [calicheamicins and pyrrolobenzodiazepine dimers (PBDs), and topoisomerase I inhibitors (SN-38 and DXd)]. ADCs with other payloads including tubulysin (an anti-mitotic agent), duocarmycins (DNA alkylating agents), PNU-159682 (a topoisomerase I inhibitor), and goitrogens (an RNA polymerase I inhibitor), are currently being evaluated in preclinical and clinical studies. The linker allows the cytotoxic payload to attach to the antibody until it is internalized and translocated to specific cellular compartments (Tsuchikama et al., 2024). Cleavable and non-cleavable linkers are the two main types of ligands. Non-cleavable linkers are based on thioether or maleimidocaproyl and require lysosomal proteolysis, whereas cleavable linkers are peptide-, disulfide-, or pH-dependent and are released through hydrolysis, enzymatic cleavage, or reduction (Lu et al., 2016; Kostova et al., 2021).

Although ADCs are highly efficacious compared with conventional small-molecule chemotherapy agents, their adverse reactions should not be ignored. Several clinical trials of ADCs have shown a variety of ocular adverse events (AEs) during ADCs treatment. In 2015, Eaton et al. conducted a comprehensive review of ocular adverse reactions to ADCs in human clinical trials, in which the most common ocular adverse effects included blurred vision, keratitis, dry eyes, microcystic epithelial changes, and corneal deposits (Eaton et al., 2015; Raheem et al., 2023). The exact process of ADCs-induced ocular toxicity is unknown, potential reasons include: First, ocular tissues may sustain harm as a result of linker-carrier instability, which causes the circulating carrier to release prematurely. Second, bystander effect: target antigen-negative cells nearby are subjected to harmful effects from the free payload of the ADCs. Third, normal cellular uptake: through receptor-dependent and non-receptor-dependent (non-specific endocytosis) methods, normal cells may take up and transport intact ADC medicines, which could be harmful to the eyes. Fourth, drug particularity: certain ADCs such as the medenosine derivative DM4 and auristatin analogs (such as monomethyl auristatin F MMAF and monomethyl auristatin E MMAE), can directly cause cytotoxicity in corneal epithelial cells, leading to apoptosis and microcystic corneal epithelial alterations. Therefore, with the advent of ADCs, any ocular toxicity caused by ADCs should be of concern.

Nevertheless, comprehensive studies on ADCs-related ocular AEs are still lacking. The US FDA Adverse Event Reporting System (FAERS) database is used to help the FDA monitor the safety of post-marketing medications, including all adverse event information collected by the FDA. Pharmacovigilance is an important way to discover the association between post-marketing drugs and AEs (Song et al., 2023). Mining based on FAERS massive data can better discover real-world safety information. In order to study the relationship between ADCs and ocular AEs as well as their influencing factors, to compare the differences in ocular AEs of different ADCs, and to provide guidance for the clinical use of medication, the present study conducted an in-depth analysis of ocular AEs of post-marketing ADCs using FAERS real-world data.

## 2 Data and methods

### 2.1 Data sources

The FDA’s post-marketing safety surveillance program for drug and therapeutic biologic products is supported by voluntary reports that are incorporated in the FAERS database from a variety of sources, including patients, pharmacists, healthcare professionals, and pharmaceutical corporations. It includes a variety of data, such as patient demographics, drug utilization, adverse reaction information, reporting sources, duration of therapy, drug indications, and patient outcomes. There are seven different types of data documents in the FAERS data files: DRUG (drug information), OUTC (patient outcomes), RPSR (report sources), THER (therapy start and end dates for reported drugs), INDI (indications for drug administration), and DEMO (demographic and administrative information). In the FAERS database architecture, these files were linked together by unique identifying numbers like PRIMARYID (Unique number for identifying a FAERS report).

### 2.2 Data extraction

In this study, the following drugs were selected for research: belantamab mafodotin (BM), brentuximab vedotin (BV), enfortumab vedotin (EV), mirvetuximab soravtansine (MS), sacituzumab govitecan (SG), trastuzumab deruxtecan (TD), and trastuzumab emtansine (TE). All AEs were classified using the Medical Dictionary of Regulatory Activities (MedDRA; version 25.1), and preferred terms (PTs) were allocated according to systemic organ classes (SOCs). The five levels of MedDRA scoring from low to high are lowest-level term (LLT), preferred term (PT), high-level term (HLT), high-level group term (HLGT), and system organ class (SOC). PTs were obtained for all ocular AEs with a SOC of “ocular system disorders.” To ensure the inclusion of the latest reports, we extracted all FAERS reports recorded between Q3 2011 and Q3 2023.

**FIGURE 1:**
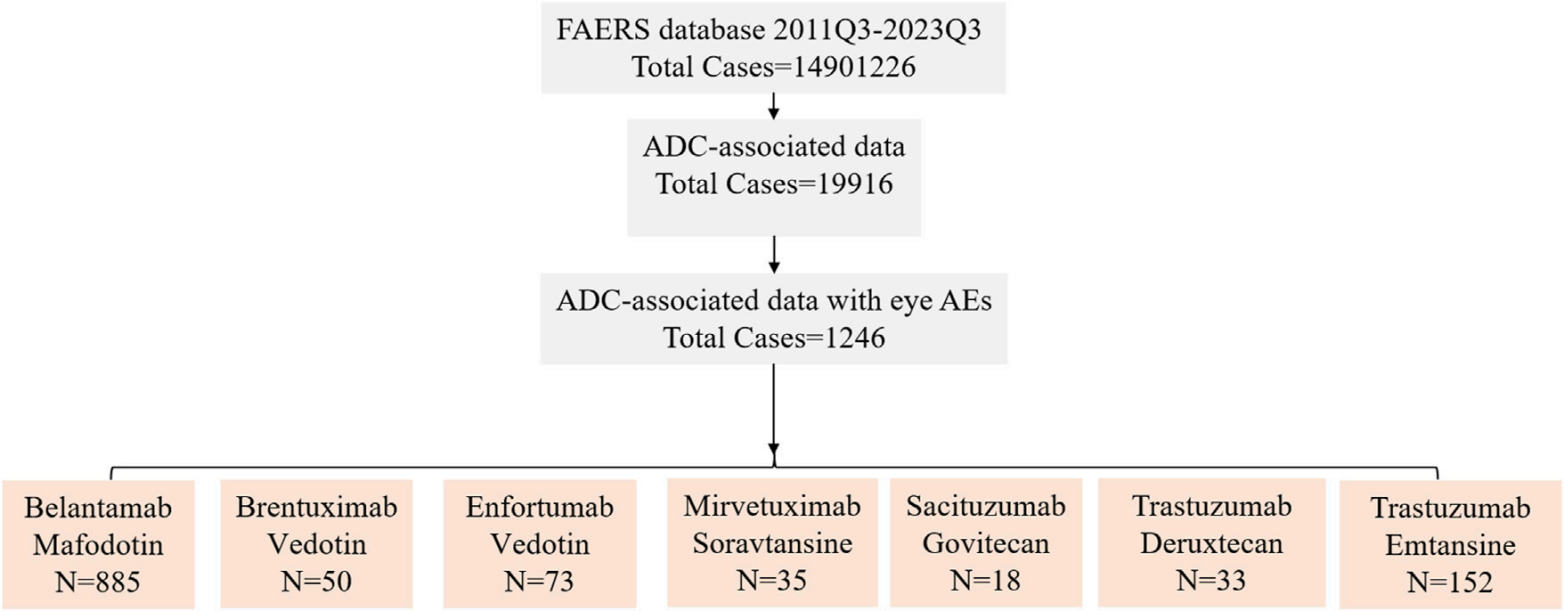
The FAERS database’s pipeline flowchart for screening ADC-associated ocular adverse events. 2011Q3 refers to the data for the third quarter of 2011, 2023Q3 refers to the data for the third quarter of 2023.

Figure 1 shows the comprehensive screening process. Two reports were deemed duplicates if they had the same adverse event, ISR number, date of delivery, medicine, indication, sex, reporting country, and age. After excluding probable ocular AEs that are attributable to concurrent drugs and drug interactions, the remaining reports were further screened by designating the principal suspect (PS) as ADCs. Following the aforementioned deduplication procedure, additional analysis was performed on the remaining reports.

### 2.3 Data analysis

Proportional disequilibrium and Bayesian methods are critical analytical tools used in the field of pharmacovigilance. The proportional disequilibrium method involves a comparison of the proportion of AEs occurring between the target drug and all other drugs. This analysis includes the proportional reported ratio (PRR) and the reported odds ratio (ROR) (Sakaeda et al., 2013). On the other hand, the Bayesian approach employs two prominent algorithms: the Bayesian confidence propagation neural network (BCPNN) and the multiple Gamma Poisson Shrinker (MGPS) (Song et al., 2020). In order to enhance the credibility of the correlation analysis between drugs and AEs, four distinct algorithms were used in this study: ROR, PRR, BCPNN, and MGPS. These algorithms were employed to quantify the correlation between ADCs and ocular AEs, respectively. The formulas and signal detection criteria for these four algorithms are listed in Table 1, based on the four-cell table for the proportional disequilibrium method (Supplementary Table S1) and the Bayesian method (Shu et al., 2022a). In general, higher algorithm values signify a more pronounced signal, indicating a stronger correlation between the drug and the occurrence of AEs. The time to occurrence of AEs was calculated using the following formula: Time to occurrence of AEs=Date of AEs − Date of initiation of ADCs . Data entry errors (EVENT_DT preceded START_DT) or inaccuracies were excluded (Shu et al., 2022b; Chen et al., 2024). The median value was employed to describe the time required for AEs to occur. We analyzed the ocular AEs signals associated with ADCs and the corresponding PT mortality.

**TABLE 1:** Four major algorithms used for signal detection.

Algorithms	Equation	Criteria
ROR	ROR=ad/b/c	lower limit of 95% CI > 1, N ≥ 3
	$95\%\text{CI}=e^{\ln\left(\text{ROR}\right)\pm 1.96(1/a+1/b+1/c+1/d{)}^{\wedge }0.5}$	
PRR	$\text{PRR}=a\left(c+d\right)/c/\left(a+b\right)$	PRR≥2, χ^2^ ≥ 4, N ≥ 3
	$\chi^{2}=\left[\left(\text{ad}-\text{bc}{)}^{\wedge }2\right.\right]\left(a+b+c+d\right)/\left[\left(a+b\right)\;\left(c+d\right)\;\left(a+c\right)\;\left(b+d\right)\right]$	
BCPNN	$\text{IC}=\log_{2}a\left(a+b+c+d\right)\;\left(a+c\right)\;\left(a+b\right)$	IC025 > 0
	$95\%\text{CI}=E\left(\text{IC}\right)\;\pm \;2V(\text{IC}{)}^{\wedge }0.5$	
MGPS	$\text{EBGM}=a\left(a+b+c+d\right)/\left(a+c\right)/\left(a+b\right)$	EBGM05 > 2
	$95\%\text{CI}=e^{\ln\left(\text{EBGM}\right)\pm 1.96(1/a+1/b+1/c+1/d{)}^{\wedge }0.5}$	

Equation: a, number of reports containing both the target drug and target adverse drug reaction; b, number of reports containing other adverse drug reaction of the target drug; c, number of reports containing the target adverse drug reaction of other drugs; d, number of reports containing other drugs and other adverse drug reactions. 95%CI, 95% confidence interval; N, the number of reports; χ2, chi-squared; IC, information component; IC025, the lower limit of 95% CI, of the IC; E(IC), the IC, expectations; V(IC), the variance of IC; EBGM, empirical Bayesian geometric mean; EBGM05, the lower limit of 95% CI of EBGM.

### 2.4 Statistical analysis

All statistical analyses and data mining were performed using R software (version 4.1.2) and Microsoft Excel 2019. The chi-square test was used to compare the ADCs-related ocular AEs between the serious and non-serious groups.

## 3 Results

### 3.1 Ocular AEs among ADCs users in FAERS from 2011 to 2023

**FIGURE 2:**
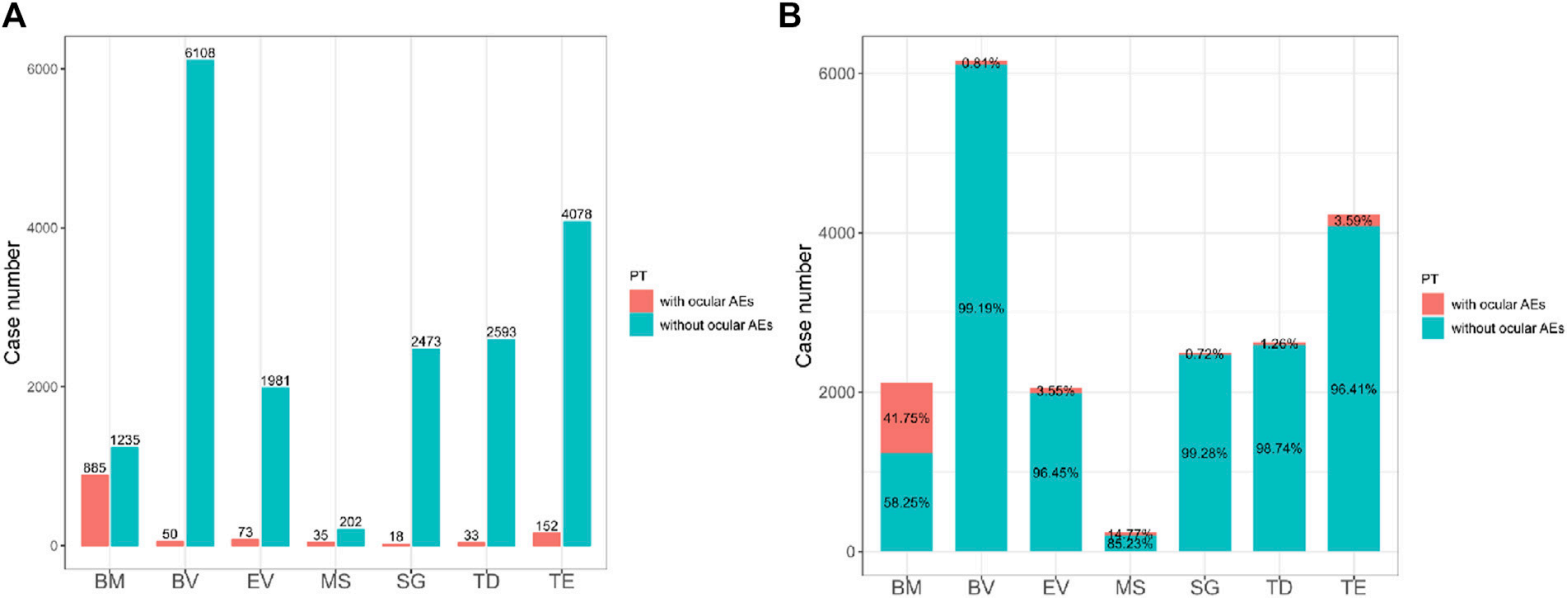
**(A)** The bar chart above shows the number of reported ocular adverse events and without ocular adverse events of different ADCs in the FAERS database from 2011 Q3 to 2023 Q3. **(B)** The proportional bar chart below shows the percentage of different ADCs ocular adverse events and without ocular adverse events in the FAERS database from 2011 Q3 to 2023 Q3. BM, belantamab mafodotin; BV, brentuximab vedotin; EV, enfortumab vedotin; MS, mirvetuximab soravtansine; SG, sacituzumab govitecan; TD, trastuzumab deruxtecan; TE, trastuzumab emtansine.

Using the FAERS database, we first investigated the incidence of ocular AEs in patients receiving ADCs between Q3 2011 and Q3 2023. A total of 14,901,226 AE reports were included in the FAERS database. After excluding duplicate reports, 1,246 cases of ADCs-related ocular AEs were identified. Ocular AEs accounted for only a small fraction of the total number of adverse reactions reported for all ADCs (6.26%, 1,246/19,916) (Table 2). The number of cases of ocular AEs has increased significantly in recent years, which is a cause for concern. However, ADCs differed in the frequency of ocular AEs. These included cases associated with BM (885 cases), BV (50 cases), EV (73 cases), MS (35 cases), SG (18 cases), TD (33 cases) and TE (152 cases). The two medications with the highest incidence of ocular AEs were BM [41.75% (885/2,120)] and MS [14.77% (35/237)]. The incidence of ocular AEs for TE, EV, BV, TD, and SG was comparatively low at 3.59% (152/4,230), 3.55% (73/2,054), 1.26% (50/2,626), 0.81% (33/6,158), and 0.72% (18/2,491), respectively (Figure 2). Overall, the percentage of ocular AEs in different ADCs treatments indicated that ocular AEs account for a non-negligible portion of potential AEs in ADCs.

**TABLE 2:** The counts of reports with ADCs related Ocular AEs yearly from 2011Q3 to 2023Q3.

Years	Ocular AEs	Non Ocular AEs	Total
2011	0	16	16
2012	4	135	139
2013	10	452	462
2014	13	692	705
2015	31	938	969
2016	24	817	841
2017	18	818	836
2018	15	885	900
2019	11	1,103	1,114
2020	49	1,497	1,546
2021	284	2,148	2,432
2022	518	4,284	4,802
2023	269	4,885	5,154
Total	1,246	18,670	19,916

### 3.2 Clinical characteristics of ADC-related ocular AEs

We obtained 1,246 cases of ocular AEs with the “primary suspect” of ADCs from the FAERS database. Table 3 provides the clinical characteristics of the patients. Among the reported ocular AEs associated with ADCs, females (32.5%) were at higher risk than males (23.27%), and sex information was not available for 44.22% of the cases (551/1,246). Among ocular AEs, the proportion of females treated with BM, EV, BV, TD, TE, and SG was 21.5% (190/885), 24.7% (18/73), 40.0% (20/50), 66.7% (22/33), 90.1% (137/152), and 100% (18/18), respectively. Nevertheless, the percentage of males presenting with ocular AEs for TE, TD, BM, BV, and EV was 2.0% (3/152), 6.1% (2/33), 23.7% (210/885), 46.0% (23/50), and 71.2% (52/73) in that order. Of the 467 AE reports available, patients aged 18∼64, 65∼85, and >85 years accounted for 221 (17.74%), 231 (18.54%), and 15 (1.2%) cases, respectively. Physicians reported the greatest number of cases (35.41%, 656/1,246), followed by consumers (26.57%, 331/1,246) and health professionals (14.21%, 177/1,246). Cases with a fatal outcome accounted for 15.57% (194/1,246). The United States accounted for 75.52% (N = 941) of the reporting countries.

**TABLE 3:** Characteristics of ocular toxicity correlated with ADCs.

Characteristics	Belantamab Mafodotin	Brentuximab Vedotin	Enfortumab Vedotin	Mirvetuximab Soravtansine	Sacituzumab Govitecan	Trastuzumab Deruxtecan	Trastuzumab Emtansine
Gender	N = 885	N = 50	N = 73	N = 35	N = 18	N = 33	N = 152
Female	190 (21.5%)	20 (40.0%)	18 (24.7%)	0 (0%)	18 (100%)	22 (66.7%)	137 (90.1%)
Male	210 (23.7%)	23 (46.0%)	52 (71.2%)	0 (0%)	0 (0%)	2 (6.1%)	3 (2.0%)
Unknown	485 (54.8%)	7 (14.0%)	3 (4.1%)	35 (100%)	0 (0%)	9 (27.3%)	12 (7.9%)
Age							
18–64	99 (11.2%)	26 (52.0%)	8 (11.0%)	4 (11.4%)	10 (55.6%)	12 (36.4%)	62 (40.8%)
65–85	173 (19.5%)	5 (10.0%)	38 (52.1%)	1 (2.9%)	1 (5.6%)	4 (12.1%)	9 (5.9%)
>85	9 (1.0%)	1 (2.0%)	4 (5.5%)	1 (2.9%)	0 (0%)	0 (0%)	0 (0%)
Unknown	604 (68.2%)	18 (36.0%)	23 (31.5%)	29 (82.9%)	7 (38.9%)	17 (51.5%)	81 (53.3%)
Weight							
<50 kg	1 (0.1%)	1 (2.0%)	3 (4.1%)	0 (0%)	0 (0%)	0 (0%)	3 (2.0%)
>100 kg	5 (0.6%)	0 (0%)	2 (2.7%)	0 (0%)	1 (5.6%)	0 (0%)	2 (1.3%)
50∼100 kg	44 (5.0%)	9 (18.0%)	12 (16.4%)	0 (0%)	11 (61.1%)	5 (15.2%)	39 (25.7%)
Unknown	835 (94.4%)	40 (80.0%)	56 (76.7%)	35 (100%)	6 (33.3%)	28 (84.8%)	108 (71.1%)
Reporters							
Consumer	218 (24.6%)	10 (20.0%)	31 (42.5%)	14 (40.0%)	1 (5.6%)	4 (12.1%)	53 (34.9%)
Other Health-Professional	0 (0%)	10 (20.0%)	0 (0%)	0 (0%)	0 (0%)	0 (0%)	20 (13.2%)
Physician	526 (59.4%)	19 (38.0%)	22 (30.1%)	11 (31.4%)	11 (61.1%)	16 (48.5%)	51 (33.6%)
Pharmacist	7 (0.8%)	5 (10.0%)	9 (12.3%)	1 (2.9%)	3 (16.7%)	7 (21.2%)	16 (10.5%)
Lawyer	0 (0%)	0 (0%)	0 (0%)	0 (0%)	0 (0%)	0 (0%)	0 (0%)
Health-Professional	133 (15.0%)	6 (12.0%)	11 (15.1%)	9 (25.7%)	3 (16.7%)	6 (18.2%)	9 (5.9%)
Unknown	1 (0.1%)	0 (0%)	0 (0%)	0 (0%)	0 (0%)	0 (0%)	3 (2.0%)
Outcomes							
Congenital Anomaly	0 (0%)	0 (0%)	0 (0%)	0 (0%)	0 (0%)	0 (0%)	0 (0%)
Death	175 (15.5%)	4 (5.4%)	5 (5.7%)	0 (0%)	3 (12.0%)	2 (5.3%)	5 (2.9%)
Disability	14 (1.2%)	9 (12.2%)	0 (0%)	0 (0%)	0 (0%)	2 (5.3%)	8 (4.6%)
Hospitalization - Initial or Prolonged	89 (7.9%)	17 (23.0%)	13 (14.8%)	0 (0%)	4 (16.0%)	2 (5.3%)	30 (17.1%)
Life-Threatening	3 (0.3%)	6 (8.1%)	0 (0%)	0 (0%)	0 (0%)	0 (0%)	1 (0.6%)
Other Serious (Important Medical Event)	587 (52.0%)	35 (47.3%)	46 (52.3%)	2 (5.7%)	13 (52.0%)	9 (23.7%)	81 (46.3%)
Required Intervention to Prevent	0 (0%)	0 (0%)	0 (0%)	0 (0%)	0 (0%)	0 (0%)	1 (0.6%)
Unknown	260 (23.0%)	3 (4.1%)	24 (27.3%)	33 (94.3%)	5 (20.0%)	23 (60.5%)	49 (28.0%)
Reporting Country							
The United States	753 (85.1%)	14 (28.0%)	40 (54.8%)	35 (100%)	1 (5.6%)	22 (66.7%)	76 (50.0%)
French	42 (4.7%)	0 (0%)	3 (4.1%)	0 (0%)	7 (38.9%)	4 (12.1%)	4 (2.6%)
Spain	36 (4.1%)	2 (4.0%)	0 (0%)	0 (0%)	2 (11.1%)	0 (0%)	7 (4.6%)
Austria	11 (1.2%)	1 (2.0%)	0 (0%)	0 (0%)	0 (0%)	0 (0%)	2 (1.3%)
China	3 (0.3%)	1 (2.0%)	0 (0%)	0 (0%)	0 (0%)	0 (0%)	1 (0.7%)
Germany	6 (0.7%)	0 (0%)	1 (1.4%)	0 (0%)	1 (5.6%)	1 (3.0%)	7 (4.6%)
The United Kingdom	5 (0.6%)	0 (0%)	0 (0%)	0 (0%)	0 (0%)	0 (0%)	7 (4.6%)
Greece	5 (0.6%)	0 (0%)	0 (0%)	0 (0%)	0 (0%)	0 (0%)	0 (0%)
Italy	8 (0.9%)	4 (8.0%)	0 (0%)	0 (0%)	1 (5.6%)	0 (0%)	2 (1.3%)
Other countries	16 (1.8%)	28 (56.0%)	29 (39.7%)	0 (0%)	6 (33.3%)	6 (18.2%)	45 (29.6%)
Unknown	0 (0%)	0 (0%)	0 (0%)	0 (0%)	0 (0%)	0 (0%)	1 (0.7%)

N, number of cases with ocular AEs, associated with the target drug.

### 3.3 Disproportionality analysis for ADC-related ocular AEs

The signal values and associations between ADCs and ocular AEs are shown in Table 4. BM (N = 885, ROR = 24.00, 95% CI [22.76∼25.30], PRR = 18.3, X^2^ = 30,354.27), MS (N = 35, ROR = 9.65, 95% CI [7.20∼12.94], PRR = 8.6, X^2^ = 347.16), TE (N = 152, ROR = 1.22, 95% CI [1.07∼1.40], PRR = 1.22, X^2^ = 8.55) had significant signal values. Among these, BM exhibited the strongest association with the ocular system compared to the other ADCs. In contrast, SG (N = 18, ROR = 0.22, 95% CI [0.15∼0.32], PRR = 0.22, X^2^ = 75.65) showed fewer safety concerns for the ocular system.

**TABLE 4:** Safety adverse events among different ADC drugs.

Drug	ADC-associated AEs n	ADC-associated ocular AEs n	ADC-associated ocular AEs as PSn	ROR (95%Cl)	PRR (X2)	EBGM (EBGM05)	IC (IC025)
Belantamab Mafodotin	5,610	1847	885	24 (22.76–25.3)	18.3 (30,354.27)	18.15 (17.36)	4.18 (2.52)
Brentuximab Vedotin	19,710	66	50	0.23 (0.18–0.29)	0.23 (173.2)	0.23 (0.19)	−2.12 ( −3.79)
Enfortumab Vedotin	6,517	99	73	1.09 (0.89–1.33)	1.09 (0.71)	1.09 (0.92)	0.12 ( −1.54)
Mirvetuximab Soravtansine	367	51	35	9.65 (7.2–12.94)	8.6 (347.16)	8.59 (6.73)	3.1 (1.43)
Sacituzumab Govitecan	8,934	27	18	0.22 (0.15–0.32)	0.22 (75.65)	0.22 (0.16)	−2.18 ( −3.85)
Trastuzumab Deruxtecan	6,994	37	33	0.36 (0.26–0.5)	0.37 (41.04)	0.37 (0.28)	−1.45 ( −3.11)
Trastuzumab Emtansine	11,808	213	152	1.22 (1.07–1.4)	1.22 (8.55)	1.22 (1.09)	0.29 ( −1.38)

a, the number of reports; PS, primary suspect; ROR, reporting odds ratio; PRR, proportional reporting ratio; χ^2^, chi-squared; IC, information component; IC025, the lower limit of 95% CI of the IC; EBGM, empirical Bayesian geometric mean; EBGM05, the lower limit of 95% CI of EBGM.

A total of 212 positive signals at the PT level were identified (Supplementary Table S2). The PT signals of BM, TE, MS, EV, BV, TD, and SG involved 111 signals (ROR range: 2.33∼38,373.43), 42 signals (ROR range: 1.67∼3,418.52), 21 signals (ROR range: 3.73∼618.05), 16 signals (ROR range: 1.66∼59.61), 10 signals (ROR range: 6.23∼48.19), 9 signals (ROR range: 2.76∼78.67) and 3 signals (ROR range: 3.38∼7.13), respectively.

**FIGURE 3:**
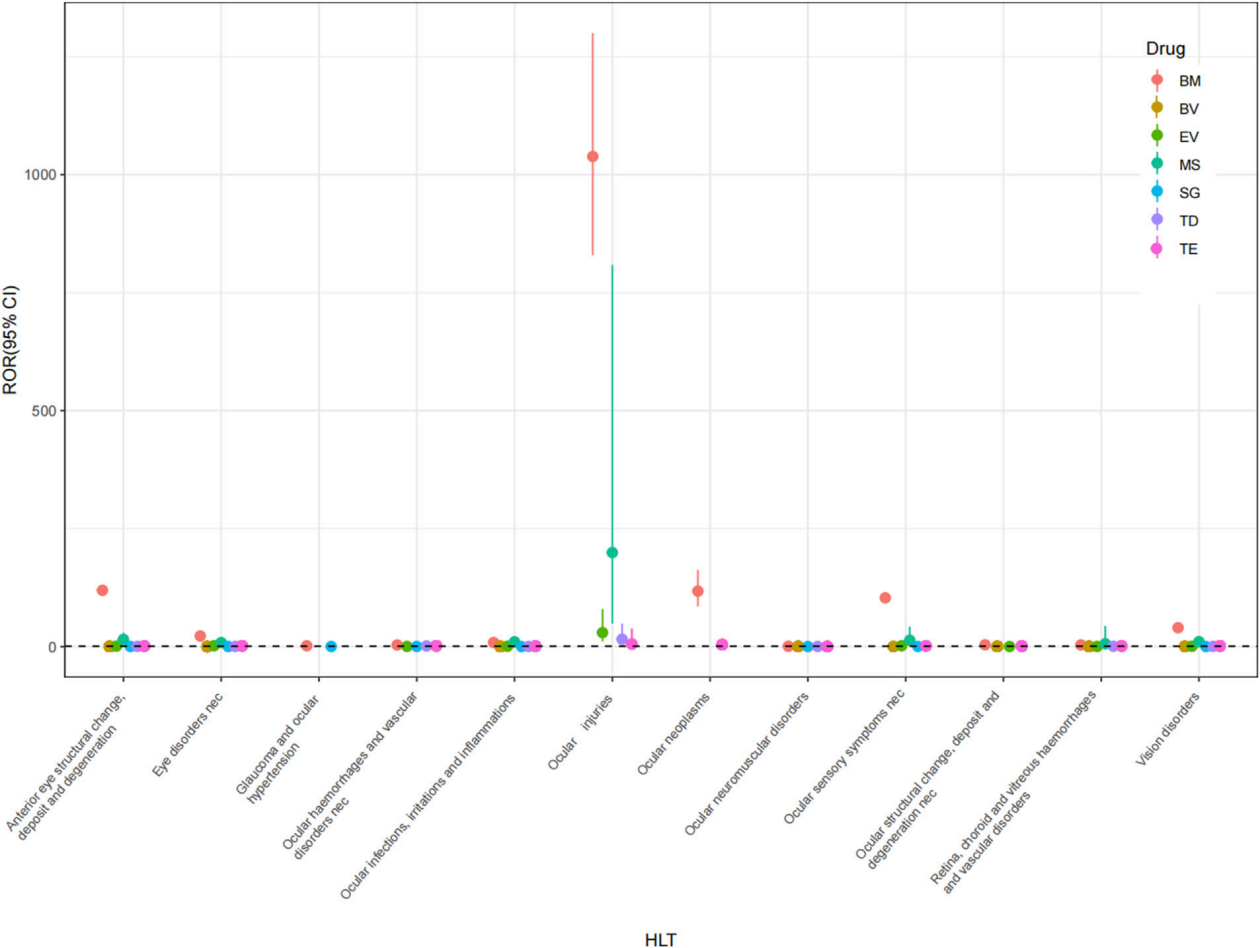
Forest plots of ICs under HLT classification of different ADCs. HLT, high-level term; BM, belantamab mafodotin; BV, brentuximab vedotin; EV, enfortumab vedotin; MS, mirvetuximab soravtansine; SG, sacituzumab govitecan; TD, trastuzumab deruxtecan; TE, trastuzumab emtansine.

Utilizing the HLGT, AEs at the PT level were clustered into the subsequent grading corresponding to the ocular-related disorders. The ocular AE signals at the HLGT level were 12, 11, 8, 7, 6, 5, and 2 for BM, TE, TD, MS, EV, BV, and SG, respectively (Figure 3).

The ocular AE signals of BM include anterior eye structural change, deposit and degeneration; vision disorders; eye disorders nec; ocular structural change, deposit and degeneration nec; ocular infections, irritations and inflammations; ocular sensory symptoms nec; retina, choroid and vitreous haemorrhages and vascular disorders; ocular haemorrhages and vascular disorders nec; ocular injuries; ocular neoplasms; ocular neuromuscular disorders; glaucoma and ocular hypertension. The ocular AE signals of TE include ocular infections, irritations and inflammations; vision disorders; anterior eye structural change, deposit and degeneration; eye disorders nec; retina, choroid and vitreous haemorrhages and vascular disorders; ocular structural change, deposit and degeneration nec; ocular haemorrhages and vascular disorders nec; ocular injuries; ocular neoplasms; ocular neuromuscular disorders and ocular sensory symptoms nec. The ocular AE signals of MS include ocular infections, irritations and inflammations; anterior eye structural change, deposit and degeneration; eye disorders nec; vision disorders; ocular injuries; ocular sensory symptoms nec; retina, choroid and vitreous haemorrhages and vascular disorders. The ocular AE signals of EV include eye disorders nec; ocular infections, irritations and inflammations; vision disorders; ocular injuries; ocular sensory symptoms nec; retina, choroid and vitreous haemorrhages and vascular disorders. The ocular AE signals of BV include vision disorders; anterior eye structural change, deposit and degeneration; ocular infections, irritations and inflammations; ocular structural change, deposit and degeneration nec; retina, choroid and vitreous haemorrhages and vascular disorders. The ocular AE signals of TD include ocular infections, irritations and inflammations; anterior eye structural change, deposit and degeneration; eye disorders nec; ocular haemorrhages and vascular disorders nec; ocular injuries; ocular neuromuscular disorders; retina, choroid and vitreous haemorrhages and vascular disorders; vision disorders. The ocular AE signals of SG include eye disorders nec and vision disorders.

**FIGURE 4:**
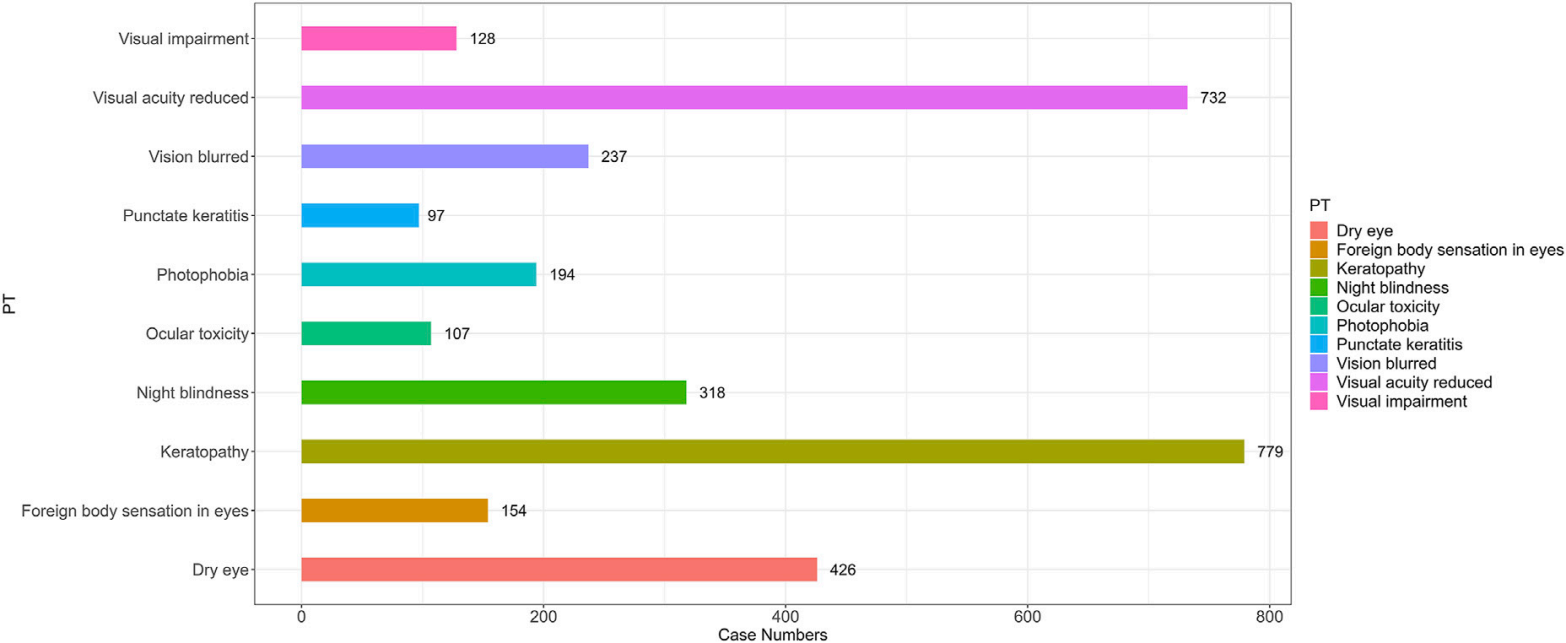
The number of reported cases of the first ten types of ADC related ocular AEs under different ADC treatment strategies. PT, preferred term.

To better understand the clinical characteristics of ocular AE, we further explored the top ten most frequently reported ocular AE after ADCs therapy. The ten most common ADC-related ocular AE signals are keratopathy [N = 779, ROR = 1,273.52, 95%CI (1,129.26∼1,436.21), PRR = 1,258.34, X^2^ = 335,325.22], visual acuity reduced [N = 733, ROR = 22.83, 95%CI (21.2∼24.58), PRR = 22.58, X^2^ = 14,623.85], dry eye [N = 435, ROR = 9.69, 95%CI (8.81∼10.66), PRR = 9.63, X^2^ = 3,318.75], night blindness [N = 319, ROR = 259.87, 95%CI (228.23∼295.89), PRR = 258.6, X^2^ = 58,709.26], vision blurred [N = 244, ROR = 1.78, 95% CI (1.57∼2.02), PRR = 1.78, X^2^ = 83.54], photophobia [N = 194, ROR = 10.45, 95%CI (9.07∼12.05), PRR = 10.43, X^2^ = 1,627.88], foreign body sensation in eyes [N = 154, ROR = 23.35, 95%CI (19.88∼27.42), PRR = 23.29, X^2^ = 3,173.32], ocular toxicity [N = 107, ROR = 144.62, 95%CI (117.3∼178.32), PRR = 144.39, X^2^ = 12,487.36], punctate keratitis [N = 98, ROR = 126.21, 95%CI (101.66∼156.69), PRR = 126.02, X^2^ = 10,195.84], eye disorder [N = 93, ROR = 2.71, 95%CI (2.21∼3.32), PRR = 2.71, X^2^ = 99.84] (Figure 4; Table 5).

**TABLE 5:** The signal values of the first ten types of ADC related ocular AEs under different ADCs treatment strategies.

Preferred term (PTs)	Report number	ROR (95%Cl)	PRR (X2)	EBGM (EBGM05)	IC (IC025)
Keratopathy	779	1,273.52 (1,129.26–1,436.21)	1,258.34 (335,325.22)	431.79 (390.46)	8.75 (7.09)
Visual acuity reduced	733	22.83 (21.2–24.58)	22.58 (14,623.85)	21.86 (20.55)	4.45 (2.78)
Dry eye	435	9.69 (8.81–10.66)	9.63 (3,318.75)	9.51 (8.78)	3.25 (1.58)
Night blindness	319	259.87 (228.23–295.89)	258.6 (58,709.26)	185.75 (166.63)	7.54 (5.87)
Vision blurred	244	1.78 (1.57–2.02)	1.78 (83.54)	1.78 (1.6)	0.83 ( −0.83)
Photophobia	194	10.45 (9.07–12.05)	10.43 (1,627.88)	10.28 (9.13)	3.36 (1.7)
Foreign body sensation in eyes	154	23.35 (19.88–27.42)	23.29 (3,173.32)	22.53 (19.69)	4.49 (2.83)
Ocular toxicity	107	144.62 (117.3–178.32)	144.39 (12,487.36)	118.52 (99.46)	6.89 (5.22)
Punctate keratitis	98	126.21 (101.66–156.69)	126.02 (10,195.84)	105.87 (88.34)	6.73 (5.06)
Eye disorder	93	2.71 (2.21–3.32)	2.71 (99.84)	2.7 (2.28)	1.43 ( −0.23)

PTs, preferred terms; ROR, reporting odds ratio; PRR, proportional reporting ratio; χ^2^: chi-squared; IC: information component; IC025: the lower limit of 95% CI, of the IC; EBGM, empirical Bayesian geometric mean; EBGM05, the lower limit of 95% CI, of EBGM.

**FIGURE 5:**
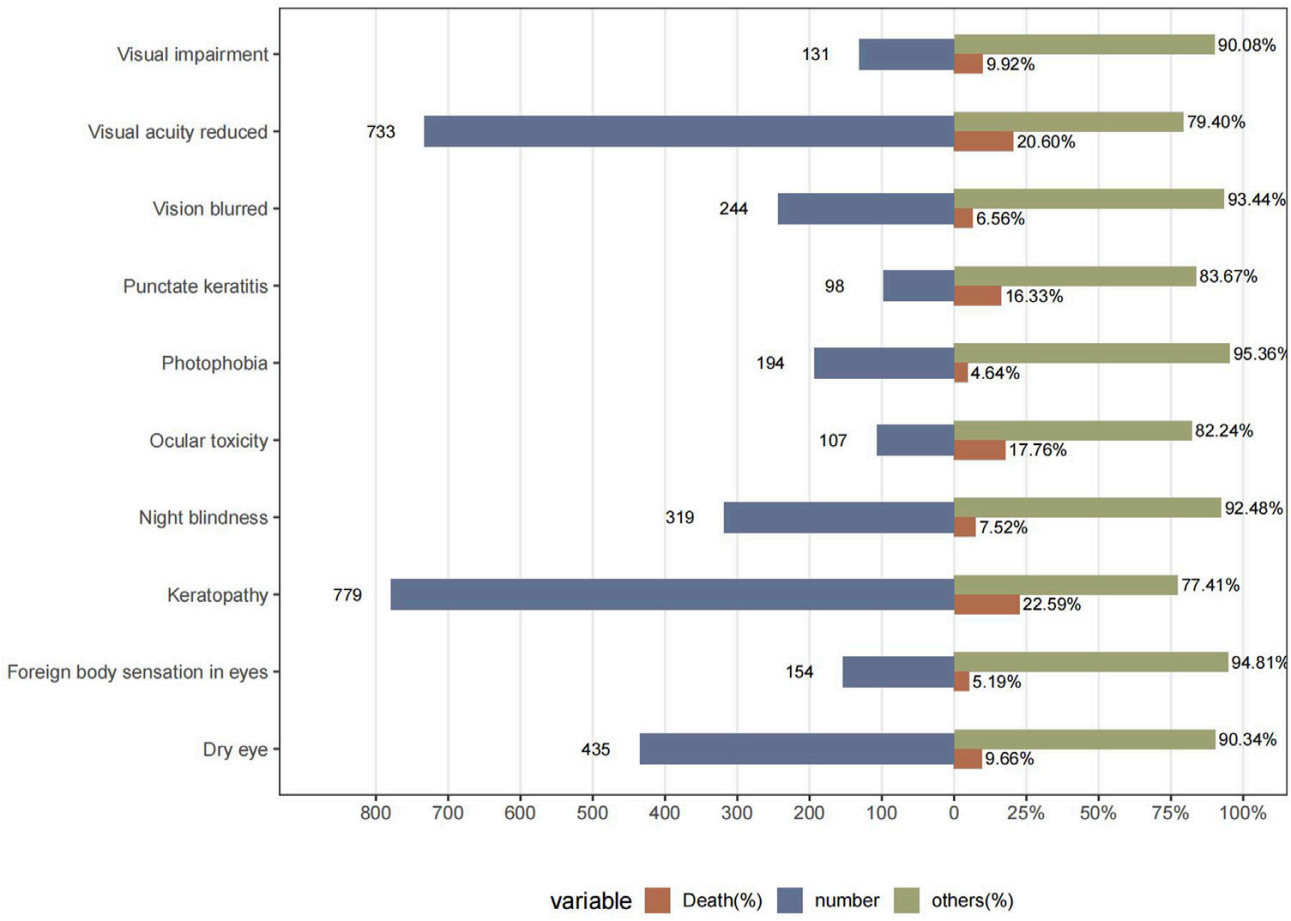
Death cases and their proportion in ADCs concomitantly with ocular AEs.

To help clinicians detect highly ocular AEs, we further calculated the mortality rates of different AEs (number of deaths reported/number of AEs reported) after ADCs therapy (Figure 5). The results showed that the mortality rates of two AEs, visual acuity reduced and keratopathy, were more than 20%. The mortality rates of ADCs with concomitant ocular system signals were 17.76% for ocular toxicity, 16.33% for punctate keratitis, 9.92% for visual impairment, 9.66% for dry eye, 7.52% for night blindness, 6.56% for vision blurred, 5.19% for foreign body sensation in eyes, and 4.64% for photophobia (Figure 5).

### 3.4 Time to onset of ocular adverse event

**FIGURE 6:**
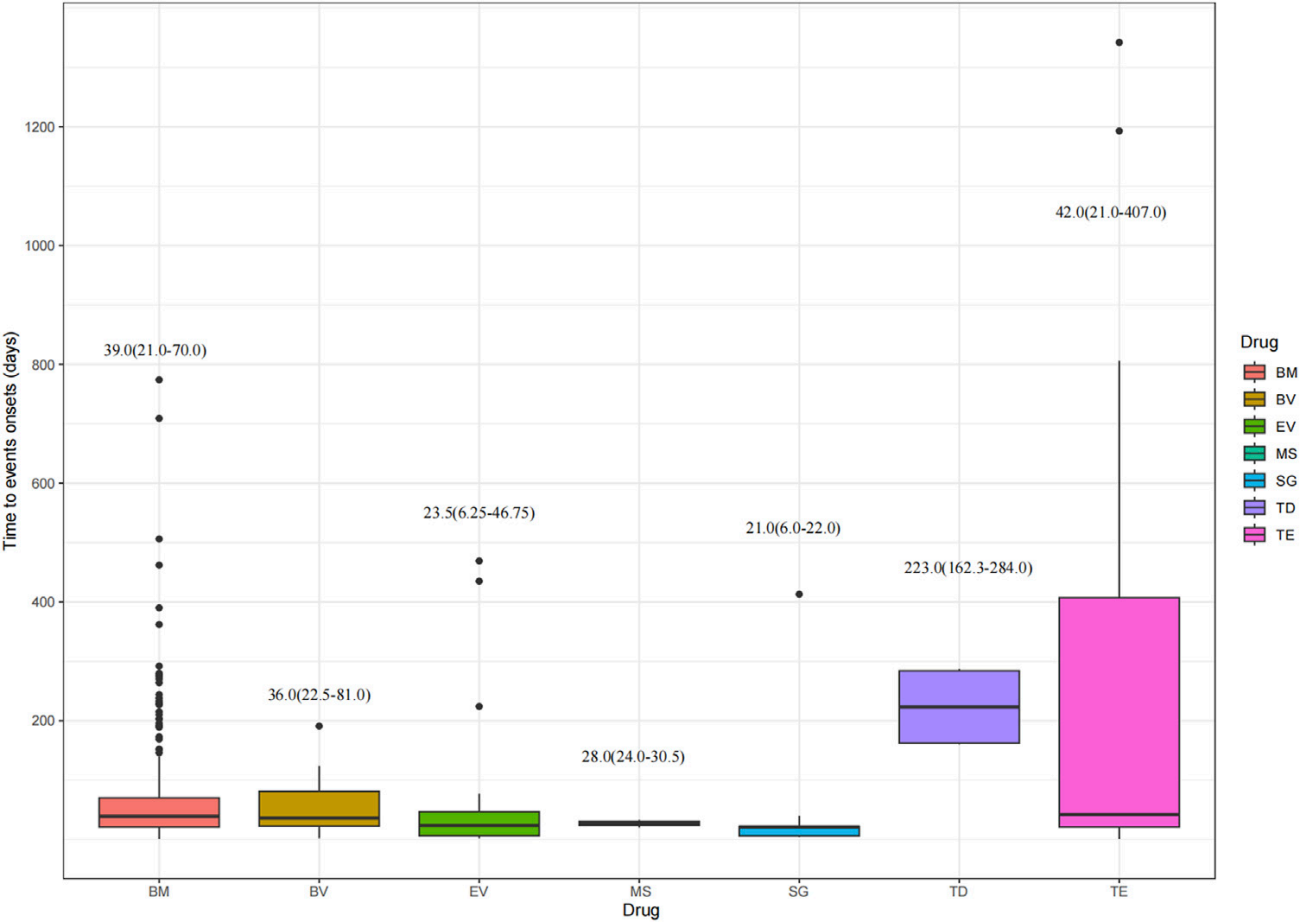
Time to onset of ocular adverse events. BM, belantamab mafodotin; BV, brentuximab vedotin; EV, enfortumab vedotin; MS, mirvetuximab soravtansine; SG, sacituzumab govitecan; TD, trastuzumab deruxtecan; TE, trastuzumab emtansine.

Figure 6 depicts the time to onset of ocular AEs for the various ADCs. The median time to onset was earlier for SG at 21 days (IQR: 6∼22 days). Conversely, TD had the longest time to onset of 223 days (IQR: 162.25∼284 days). TE presented the widest range, with a median time of 42 days (IQR: 21∼407 days). The median time for BM, BV, EV, and MS were 39 days (IQR: 21∼70 days), 36 days (IQR: 22.5∼81 days), 23.5 days (IQR: 6.25∼46.75 days), 28 days (IQR: 24∼30.5 days), respectively. This study reveals the onset time of ADCs-induced ocular AEs, providing new insights into the clinical use of these drugs and their potential to trigger ocular toxicity.

### 3.5 Comparison between serious and non-serious groups for ADC-related ocular AEs

As shown in Table 6, the difference in PTs between the severe and non-severe cases of ocular AEs receiving ADCs was statistically significant. Seven AEs were more likely to be reported as serious AEs (p < 0.05), such as blindness (χ^2^ = 8.81, p < 0.05), foreign body sensation in eyes (χ^2^ = 7.83, p < 0.05), keratopathy (χ2 = 52.51, p < 0.05), night blindness (χ^2^ = 7.49, p < 0.05), photophobia (χ^2^ = 11.33, p < 0.05), vision blurred (χ^2^ = 7.51, p < 0.05), and visual acuity reduced (χ^2^ = 32.5, p < 0.05). The other 53 AEs were more inclined to be reported as non-serious AEs (p > 0.05), such as abnormal sensation in eye (χ^2^ = 1.56, p = 0.49), astigmatism (χ^2^ = 0.4, p = 0.82), blepharitis (χ^2^ = 2.65, p = 0.25), blepharospasm (χ^2^ = 1.13, p = 0.84), cataract (χ^2^ = 3.09, p = 0.11), etc.

**TABLE 6:** Comparison between the serious and non-serious groups for ADC-related ocular adverse events.

Types of PTs, n (%)	Serious case	Non-serious cases	Statistic	p-value
Abnormal sensation in eye	2 (0.34)	5 (0.13)	1.56	0.49
Anisometropia	1 (0.17)	NA (NA)	NA	NA
Astigmatism	1 (0.17)	13 (0.33)	0.4	0.82
Blepharitis	3 (0.51)	7 (0.18)	2.65	0.25
Blepharospasm	1 (0.17)	2 (0.05)	1.13	0.84
Blindness	2 (0.34)	87 (2.18)	8.81	0
Cataract	6 (1.03)	85 (2.13)	3.09	0.11
Cataract nuclear	1 (0.17)	1 (0.03)	2.48	0.61
Choroidal effusion	1 (0.17)	2 (0.05)	1.13	0.84
Conjunctival haemorrhage	1 (0.17)	3 (0.08)	0.53	1
Conjunctival oedema	1 (0.17)	1 (0.03)	2.48	0.61
Corneal defect	1 (0.17)	11 (0.28)	0.21	0.98
Corneal deposits	1 (0.17)	19 (0.48)	1.08	0.48
Corneal disorder	3 (0.51)	69 (1.73)	4.76	0.05
Corneal epithelial microcysts	7 (1.2)	68 (1.71)	0.79	0.47
Corneal epithelium defect	4 (0.68)	27 (0.68)	0	1
Corneal erosion	1 (0.17)	7 (0.18)	0	1
Corneal lesion	1 (0.17)	4 (0.1)	0.23	1
Corneal oedema	1 (0.17)	15 (0.38)	0.61	0.68
Corneal opacity	1 (0.17)	20 (0.5)	1.21	0.44
Diplopia	3 (0.51)	61 (1.53)	3.74	0.08
Dry eye	42 (7.19)	384 (9.64)	3.04	0.1
Excessive eye blinking	1 (0.17)	NA (NA)	NA	NA
Eye discharge	1 (0.17)	9 (0.23)	0.07	1
Eye disorder	11 (1.88)	82 (2.06)	0.07	0.91
Eye irritation	4 (0.68)	39 (0.98)	0.46	0.65
Eye movement disorder	1 (0.17)	2 (0.05)	1.13	0.84
Eye pain	5 (0.86)	40 (1)	0.11	0.91
Eye pruritus	1 (0.17)	20 (0.5)	1.21	0.44
Foreign body sensation in eyes	8 (1.37)	146 (3.66)	7.83	0.01
Glaucoma	1 (0.17)	4 (0.1)	0.23	1
Hypermetropia	2 (0.34)	7 (0.18)	0.72	0.73
Hypoaesthesia eye	1 (0.17)	7 (0.18)	0	1
Keratitis	13 (2.23)	73 (1.83)	0.41	0.63
Keratopathy	176 (30.14)	603 (15.14)	52.51	0
Lacrimation increased	3 (0.51)	56 (1.41)	3.12	0.12
Macular degeneration	1 (0.17)	5 (0.13)	0.08	1
Macular fibrosis	1 (0.17)	1 (0.03)	2.48	0.61
Macular oedema	1 (0.17)	3 (0.08)	0.53	1
Maculopathy	2 (0.34)	2 (0.05)	4.95	0.14
Myopia	1 (0.17)	12 (0.3)	0.3	0.89
Night blindness	24 (4.11)	294 (7.38)	7.49	0.01
Ocular discomfort	3 (0.51)	25 (0.63)	0.11	0.97
Ocular toxicity	19 (3.25)	88 (2.21)	2.3	0.17
Photophobia	9 (1.54)	185 (4.64)	11.33	0
Photopsia	1 (0.17)	3 (0.08)	0.53	1
Pinguecula	1 (0.17)	NA (NA)	NA	NA
Presbyopia	2 (0.34)	1 (0.03)	7.79	0.05
Punctate keratitis	16 (2.74)	81 (2.03)	1.17	0.35
Purtscher retinopathy	1 (0.17)	NA (NA)	NA	NA
Retinal disorder	1 (0.17)	2 (0.05)	1.13	0.84
Retinal haemorrhage	3 (0.51)	7 (0.18)	2.65	0.25
Retinopathy	1 (0.17)	1 (0.03)	2.48	0.61
Retinopathy hypertensive	1 (0.17)	1 (0.03)	2.48	0.61
Strabismus	1 (0.17)	2 (0.05)	1.13	0.84
Vision blurred	16 (2.74)	221 (5.55)	7.51	0.01
Visual acuity reduced	151 (25.86)	581 (14.58)	32.5	0
Visual impairment	13 (2.23)	115 (2.89)	0.78	0.46
Vitreous degeneration	1 (0.17)	1 (0.03)	2.48	0.61
Vitreous haemorrhage	1 (0.17)	2 (0.05)	1.13	0.84

PTs, preferred terms; n, the number of reports.

## 4 Discussion

The discovery of ADCs has revolutionized the treatment of cancer, but their underlying ocular toxicity is often overlooked. Improving pharmaceutical safety requires an assessment of the possible ocular toxicity associated with specific ADCs. By performing a retrospective pharmacovigilance analysis using FAERS data from the past 12 years, our study offers a thorough understanding. After data cleaning and deduplication, a total of 1,246 reports of ADCs-related ocular toxicity were found. This research is the first large-scale analysis of post-marketing data and investigates the relationship between ADCs and ocular toxicity.

Despite a paucity of published evidence regarding the ocular toxicity of ADCs in the preclinical literature, ocular AEs have been reported in clinical investigations (Eaton et al., 2015). In our real-world research, signs of ocular toxicity were observed with both microtubule polymerization inhibitors (BM, BV, EV, TE, and MS) and DNA-damaging agents (TD and SG). The data indicated that individuals treated with microtubule polymerization inhibitors are at higher risk of developing ocular toxicity in comparison to those treated with other ADCs.

The eye may be susceptible to toxicity due to several factors, including its inherently robust blood supply, the presence of rapidly dividing subpopulations of cells, and the abundance and variety of cell surface receptors (Renouf et al., 2012; Raheem et al., 2023). Rather than targeting antibodies or linkers, distinct payloads are the primary source of ADCs-associated ocular AEs (Tolcher, 2016). The payload of BV and EV was the tubulin inhibitor MMAE, that of BM was the tubulin inhibitor MMAF, that of TE was the tubulin inhibitor DM1, that of MS was the tubulin inhibitor DM4, and that of TD and SG was DNA damage calicheamicin derivative. Our study revealed that among these ADCs, BM was most susceptible to ocular toxicity, followed by MS, TE, EV, TD, BV, and SG, and the signal values of BM, MS, and TE were significantly higher than those of other ADCs. The reason for this is that the BM-related ocular AEs are related to its cytotoxic payload MMAF. MMAF inhibits microtubule proteins, leading to off-target apoptosis of corneal epithelial cells, which ultimately develops into microcystic corneal epithelial changes or slit lamp keratopathy (Wahab et al., 2021). The ocular toxicity of MS is related to the “off-target” effect of the DM4 payload molecule (a derivative of medenosine), which exerts an anti-disintegrative effect on intracellular Schwann cells in the corneal epithelium, leading to epithelial disruption (e.g., corneal epithelial microcysts) (Matulonis et al., 2019; Canestraro et al., 2022). This finding is consistent with the pivotal data reported in the research that associate DM1 with hepatotoxicity and thrombocytopenia, MMAF and DM4 with ocular toxicity, and MMAE with peripheral neuropathy, anemia, and neutropenia (Jaffry et al., 2023). In addition, as reported in the literature, Joanna C. Masters et al. found that ADCs containing MMAF payloads were most frequently associated with ocular toxicities, but DM4 and DM1 were also less frequently associated with ocular toxicities (Kebriaei et al., 2018; Masters et al., 2018; Mecklenburg, 2018). This result further confirms the accuracy of our study and emphasizes the reliability of the FAERS data and the viability of the study methodology.

ADCs are associated with relatively high AEs of ocular toxicity compared with traditional chemotherapy drugs, small molecule targeting drugs, and other anti-tumor drugs (Gouda and Subbiah, 2022; Atiq et al., 2023). There are currently 15 ADCs available on the global market. Three ADCs (BM, tisotumab vedotin, and MS) are required by the FDA to state notable eye-related AEs in a “Black Box” warning. In this study, we were unable to obtain AEs data for tisotumab vedotin due to its short launch period. However, the labeling of tisotumab vedotin specifies that ocular toxicity is a warning notice. In the Innova tisotumab vedotin 204 trial (Coleman et al., 2021), 138 ocular AEs occurred in 101 patients with recurrent or metastatic cervical cancer (53%), the majority of which were grade 1∼2 and confined to the ocular surface, with 26% reporting conjunctivitis and dry eye, 11% developing keratitis, grade 3 ulcerative keratitis in 2 patients (2%), and dosage reductions due to ocular toxicity in 22%. The “Black Box” warning that BM can cause changes in the corneal epithelium resulting in vision changes, including symptoms such as severe vision loss, corneal ulcer, blurred vision, and dry eyes. Our study supplemented the FDA labeling by identifying additional ocular AEs to BM, including night blindness, photophobia, foreign body sensation in eyes, punctate keratitis, corneal epithelial microcysts, cataract, diplopia, corneal cyst, lacrimation increased, corneal opacity, corneal oedema, corneal deposits, eye pruritus, astigmatism, and hypoaesthesia eye. This provides patients with more comprehensive medication warnings for clinical use. Besides, the “Black Box” warning states that MS can cause severe ocular toxicities, including visual impairment, keratopathy, dry eye, photophobia, eye pain, and uveitis. According to safety data presented at the 2022 ASCO Congress (Zhu et al., 2023), the most common adverse reactions to MS included blurred vision (63%), fatigue (58%), keratoconus (43%), and dry eye (35%). Nevertheless, our study also revealed new signals of ocular AEs caused by MS including cataract, eye irritation, corneal epithelial microcysts, pseudophakia, lacrimation increased, blepharitis, retinal haemorrhage, and blindness. Physicians should be cautioned not to overlook these new signals because of the paucity of reports.

Furthermore, ocular disorders are listed as warning notices in EV’s product labeling. Most of these events involve the cornea and include events related to dry eye such as keratitis, blurred vision, increased lacrimation, conjunctivitis, limbal stem cell deficiency, and keratopathy. New PT signals involve retinopathy hypertensive, periorbital oedema, night blindness, abnormal sensation in eye, eye discharge. Although reports of TE-related ocular AEs in clinical trials are scarce (Tsuda et al., 2016; Banerji et al., 2019) and not mentioned in drug inserts, our study identified the following new ocular PTs: retinal detachment, asthenopia, vitreous haemorrhage, scintillating scotoma. Up to this point, there have been no reported cases of ocular toxicity in patients caused by BV, TD, or SG. Ocular toxicity is a new AE signal that is not covered by these drug labels. Thus, further investigation of any potential additive optic nerve toxicity in patients treated with ADCs is needed to ascertain the true risk. Our study revealed the prevalence of various ocular toxicity risks and the time to onset of ocular AEs associated with ADCs, which are not mentioned in the package insert. Based on actual data, this study provided clinicians with a general time frame that can help distinguish between different types of ADCs.

Our analysis identified the top ten significant ocular AEs that may occur after ADCs therapy: keratopathy, visual acuity reduced, dry eye, night blindness, vision blurred, photophobia, foreign body sensation in eyes, ocular toxicity, punctate keratitis, and eye disorder. According to pivotal clinical trials (Matsumiya et al., 2021; Phase III, randomized trial of mirvetuximab soravtansine versus chemotherapy in patients with platinum-resistant ovarian cancer_ primary analysis of FORWARD I.pdf, n.d.), numerous reports of keratopathy, visual acuity reduced, dry eye, vision blurred, photophobia, and ocular toxicity have also been observed. However, there are few reports of night blindness, foreign body sensation in eyes, and punctate keratitis, suggesting that these ocular AEs were largely underestimated in clinical trials. The reason for this may be related to strict inclusion criteria and careful selection of patients for these clinical trials. The se adverse reactions are the most common adverse effects associated with ADCs and the most common reason for discontinuation. It is generally accepted that the most plausible mechanism of ocular toxicity is the development of bilateral microcystic-like epithelial alterations in the corneal periphery that move toward the corneal center, resulting in dry eyes and blurred vision (Mickevicius et al., 2023).

Compared to other types of AEs (e.g., liver damage, QT prolongation, interstitial lung disease, and thromboembolism), ocular AEs are often overlooked and may not materially affect treatment choices. However, when these ocular symptoms worsen, they are potentially life-threatening, causing serious and possibly permanent problems. Of these, the most lethal are keratopathy and visual acuity reduced. Therefore, clinicians should remind patients to monitor their symptoms following ADCs delivery to prevent the development of severe keratopathy or visual acuity reduced. If keratopathy or visual acuity reduced is suspected, appropriate screening ought to be carried out. Once the diagnosis is confirmed, ADCs should be discontinued immediately and an appropriate course of treatment should be followed.

Since serious ocular AEs are uncommon, it is extremely important from a clinical perspective to quickly recognize and distinguish between the different types of AEs. It is essential to discontinue the drugs in question as soon as possible and to take the correct management measures. The relatively short median time to onset of ocular AEs in our real-world pharmacovigilance study (except TD, which had a median time to onset of 223 days) is indicative of the rapid trajectory of ocular AEs, which warrants focused clinical consideration. The present study identified the median time to onset of ocular AEs caused by different ADCs, providing new information on the clinical usage of these medications and their potential to cause ocular AEs.

There are many obvious limitations in this retrospective study. First, it is challenging to draw causal conclusions from pharmacovigilance studies utilizing disproportionality analysis. Further research is required to verify these results. Second, the FAERS database has many intrinsic problems as a spontaneous reporting system, including missing data, redundant data, and diverse information sources. Third, the number of reported instances is significantly influenced by the longevity of the drug market. Nevertheless, our study provides a careful and rational assessment of the risk of ocular damage associated with several ADCs.

## 5 Conclusion

In conclusion, our research methodically examined the range of ocular toxicity associated ADCs and identified new AE signals for different ADCs. Physicians should focus on early identification and prophylactic measures when administering ADCs to cancer patients and be aware of safety concerns, such as dose changes owing to ocular toxicity. More research is needed to clarify the mechanisms of ocular toxicity caused by ADCs. Despite limitations, our findings encourage continued monitoring for ocular toxicity during ADCs therapy.

## Data Availability

The original contributions presented in the study are included in the article/Supplementary Material, further inquiries can be directed to the corresponding authors.
