# Supplementary material for: Ocular adverse events associated with antibody-drug conjugates in oncology: a pharmacovigilance study based on FDA adverse event reporting system (FAERS)
Source: Front Pharmacol. 2024 Aug 20;15:1425617. doi: 10.3389/fphar.2024.1425617 (PMC11368736; doi:10.3389/fphar.2024.1425617)
Supplement: Supplementary file 1 [file Table1.DOCX]

**Supplementary data:**

**Supplementary Table S1**

**Four-cell table of proportional disequilibrium method**

| Drugs | Target adverse reaction | Other adverse reactions | Total |
| --- | --- | --- | --- |
| Target drug | a | b | a+b |
| Other drugs | c | d | c+d |
| Total | a+c | b+d | n=a+b+c+d |

a: the number of reports containing both the suspect drug and the suspect adverse drug reaction; b: the number of reports containing the suspect adverse drug reaction with other medications (except the drug of interest); c: the number of reports containing the suspect drug with other adverse drug reactions (except the event of interest); d: the number of reports containing other medications and other adverse drug reactions; n: the number of all reports.

**Supplementary Table S2**

**Signal strength of ADC-associated ocular AEs at the PT level in the FAERS database.**

| Drugs | Preferred term (PT) | Report number | ROR(95%Cl) | PRR(X2) | EBGM(EBGM05) | IC(IC025) |
| --- | --- | --- | --- | --- | --- | --- |
| Belantamab Mafodotin | keratopathy | 777 | 12544.23 ( 10472.79 - 15025.38 ) | 11237.26 ( 1348972.14 ) | 1737.18 ( 1493.69 ) | 10.76 ( 9.09 ) |
|  | visual acuity reduced | 710 | 596.32 ( 547.16 - 649.89 ) | 539.64 ( 302357.7 ) | 427.55 ( 397.85 ) | 8.74 ( 7.07 ) |
|  | dry eye | 359 | 61.06 ( 54.84 - 67.99 ) | 58.17 ( 19632.72 ) | 56.6 ( 51.73 ) | 5.82 ( 4.16 ) |
|  | night blindness | 317 | 2930.61 ( 2470.17 - 3476.89 ) | 2806.07 ( 375646.94 ) | 1186.39 ( 1028.29 ) | 10.21 ( 8.54 ) |
|  | photophobia | 182 | 93.28 ( 80.27 - 108.4 ) | 91.03 ( 15521.82 ) | 87.21 ( 76.91 ) | 6.45 ( 4.78 ) |
|  | foreign body sensation in eyes | 151 | 270.73 ( 228.17 - 321.23 ) | 265.27 ( 35209.88 ) | 235.04 ( 203.7 ) | 7.88 ( 6.21 ) |
|  | vision blurred | 128 | 9.72 ( 8.16 - 11.58 ) | 9.57 ( 979.54 ) | 9.53 ( 8.23 ) | 3.25 ( 1.59 ) |
|  | ocular toxicity | 98 | 1073.35 ( 840.26 - 1371.1 ) | 1059.25 ( 68355.76 ) | 699.15 ( 569.65 ) | 9.45 ( 7.77 ) |
|  | punctate keratitis | 92 | 1518.21 ( 1159.26 - 1988.32 ) | 1499.49 ( 79629.43 ) | 867.1 ( 691.9 ) | 9.76 ( 8.08 ) |
|  | visual impairment | 90 | 5.88 ( 4.78 - 7.24 ) | 5.82 ( 359.2 ) | 5.81 ( 4.88 ) | 2.54 ( 0.87 ) |
|  | corneal epithelial microcysts | 74 | 38373.43 ( 14026.78 - 104979.16 ) | 37992.64 ( 144170.33 ) | 1949.29 ( 839.77 ) | 10.93 ( 9.23 ) |
|  | eye disorder | 74 | 19.69 ( 15.64 - 24.78 ) | 19.5 ( 1287.3 ) | 19.33 ( 15.94 ) | 4.27 ( 2.61 ) |
|  | keratitis | 68 | 220.89 ( 171.85 - 283.93 ) | 218.88 ( 13328.57 ) | 197.9 ( 160.4 ) | 7.63 ( 5.96 ) |
|  | blindness | 64 | 13.61 ( 10.63 - 17.42 ) | 13.5 ( 736.36 ) | 13.42 ( 10.91 ) | 3.75 ( 2.08 ) |
|  | corneal disorder | 62 | 313.15 ( 239.52 - 409.41 ) | 310.55 ( 16618.09 ) | 269.89 ( 215.67 ) | 8.08 ( 6.4 ) |
|  | cataract | 57 | 7.94 ( 6.11 - 10.31 ) | 7.88 ( 341.66 ) | 7.86 ( 6.32 ) | 2.97 ( 1.31 ) |
|  | diplopia | 52 | 21.37 ( 16.24 - 28.11 ) | 21.23 ( 992.3 ) | 21.02 ( 16.71 ) | 4.39 ( 2.73 ) |
|  | corneal cyst | 33 | 7563.54 ( 3618.03 - 15811.69 ) | 7530.07 ( 53234.37 ) | 1614.37 ( 871.04 ) | 10.66 ( 8.93 ) |
|  | eye pain | 31 | 4.78 ( 3.35 - 6.8 ) | 4.76 ( 91.93 ) | 4.75 ( 3.54 ) | 2.25 ( 0.58 ) |
|  | corneal epithelium defect | 31 | 900.41 ( 590.1 - 1373.88 ) | 896.67 ( 19305.66 ) | 624.45 ( 438.48 ) | 9.29 ( 7.6 ) |
|  | eye irritation | 27 | 4.37 ( 2.99 - 6.38 ) | 4.36 ( 69.76 ) | 4.35 ( 3.17 ) | 2.12 ( 0.45 ) |
|  | lacrimation increased | 24 | 6.77 ( 4.53 - 10.11 ) | 6.75 ( 117.16 ) | 6.73 ( 4.81 ) | 2.75 ( 1.08 ) |
|  | ocular discomfort | 21 | 12.87 ( 8.37 - 19.78 ) | 12.84 ( 227.81 ) | 12.76 ( 8.91 ) | 3.67 ( 2.01 ) |
|  | corneal opacity | 20 | 152.53 ( 96.79 - 240.36 ) | 152.12 ( 2795.56 ) | 141.7 ( 96.85 ) | 7.15 ( 5.47 ) |
|  | corneal toxicity | 17 | 874.8 ( 495.75 - 1543.66 ) | 872.8 ( 10388.59 ) | 612.79 ( 381.01 ) | 9.26 ( 7.55 ) |
|  | ocular surface disease | 16 | 354.07 ( 208.21 - 602.13 ) | 353.32 ( 4796.04 ) | 301.6 ( 193.41 ) | 8.24 ( 6.55 ) |
|  | corneal oedema | 14 | 52.56 ( 30.91 - 89.39 ) | 52.47 ( 689.2 ) | 51.18 ( 32.82 ) | 5.68 ( 4.01 ) |
|  | ulcerative keratitis | 13 | 45.95 ( 26.51 - 79.65 ) | 45.87 ( 558.17 ) | 44.89 ( 28.33 ) | 5.49 ( 3.82 ) |
|  | corneal deposits | 12 | 194.36 ( 107.48 - 351.45 ) | 194.05 ( 2105.68 ) | 177.38 ( 108.06 ) | 7.47 ( 5.79 ) |
|  | corneal defect | 12 | 851.16 ( 434.18 - 1668.59 ) | 849.79 ( 7195.89 ) | 601.36 ( 342.39 ) | 9.23 ( 7.51 ) |
|  | myopia | 10 | 35.76 ( 19.13 - 66.85 ) | 35.72 ( 331.68 ) | 35.12 ( 20.81 ) | 5.13 ( 3.46 ) |
|  | astigmatism | 10 | 53.55 ( 28.57 - 100.37 ) | 53.48 ( 501.94 ) | 52.15 ( 30.83 ) | 5.7 ( 4.03 ) |
|  | eye pruritus | 10 | 2.33 ( 1.25 - 4.33 ) | 2.32 ( 7.54 ) | 2.32 ( 1.38 ) | 1.22 ( -0.45 ) |
|  | cornea verticillata | 10 | 247.76 ( 128.51 - 477.68 ) | 247.43 ( 2190.43 ) | 220.93 ( 127.56 ) | 7.79 ( 6.09 ) |
|  | hypoaesthesia eye | 8 | 120.05 ( 58.83 - 244.98 ) | 119.92 ( 891.4 ) | 113.36 ( 62.41 ) | 6.82 ( 5.14 ) |
|  | optic neuropathy | 7 | 33.15 ( 15.71 - 69.98 ) | 33.12 ( 214.62 ) | 32.61 ( 17.45 ) | 5.03 ( 3.36 ) |
|  | corneal erosion | 6 | 195.74 ( 84.7 - 452.38 ) | 195.59 ( 1060.55 ) | 178.67 ( 88.64 ) | 7.48 ( 5.78 ) |
|  | hypermetropia | 6 | 49.93 ( 22.21 - 112.24 ) | 49.89 ( 280.63 ) | 48.73 ( 24.74 ) | 5.61 ( 3.93 ) |
|  | blepharitis | 6 | 12.69 ( 5.68 - 28.32 ) | 12.68 ( 64.14 ) | 12.61 ( 6.44 ) | 3.66 ( 1.99 ) |
|  | refraction disorder | 6 | 198.9 ( 86.01 - 459.94 ) | 198.74 ( 1076.32 ) | 181.29 ( 89.9 ) | 7.5 ( 5.8 ) |
|  | retinal haemorrhage | 6 | 10.33 ( 4.63 - 23.04 ) | 10.32 ( 50.25 ) | 10.27 ( 5.25 ) | 3.36 ( 1.69 ) |
|  | eye haemorrhage | 5 | 3.72 ( 1.55 - 8.96 ) | 3.72 ( 9.94 ) | 3.72 ( 1.78 ) | 1.89 ( 0.23 ) |
|  | retinal pigment epitheliopathy | 5 | 115.45 ( 46.89 - 284.28 ) | 115.37 ( 536.76 ) | 109.29 ( 51.42 ) | 6.77 ( 5.08 ) |
|  | corneal lesion | 5 | 151.1 ( 60.92 - 374.82 ) | 151 ( 694.03 ) | 140.73 ( 65.8 ) | 7.14 ( 5.44 ) |
|  | abnormal sensation in eye | 4 | 12.76 ( 4.77 - 34.12 ) | 12.76 ( 43.07 ) | 12.68 ( 5.57 ) | 3.66 ( 1.99 ) |
|  | asthenopia | 4 | 7.46 ( 2.79 - 19.91 ) | 7.45 ( 22.27 ) | 7.43 ( 3.27 ) | 2.89 ( 1.23 ) |
|  | maculopathy | 4 | 4.88 ( 1.83 - 13.03 ) | 4.88 ( 12.32 ) | 4.87 ( 2.14 ) | 2.28 ( 0.62 ) |
|  | neovascular age-related macular degeneration | 4 | 15.63 ( 5.84 - 41.8 ) | 15.62 ( 54.31 ) | 15.51 ( 6.81 ) | 3.95 ( 2.28 ) |
|  | ocular cyst | 4 | 410.95 ( 140.43 - 1202.61 ) | 410.73 ( 1362.45 ) | 342.44 ( 139.44 ) | 8.42 ( 6.66 ) |
|  | retinal detachment | 3 | 3.2 ( 1.03 - 9.93 ) | 3.2 ( 4.52 ) | 3.19 ( 1.24 ) | 1.68 ( 0.01 ) |
|  | macular oedema | 3 | 5.5 ( 1.77 - 17.09 ) | 5.5 ( 11.02 ) | 5.49 ( 2.13 ) | 2.46 ( 0.79 ) |
|  | choroidal effusion | 3 | 27.51 ( 8.81 - 85.98 ) | 27.5 ( 75.61 ) | 27.15 ( 10.47 ) | 4.76 ( 3.09 ) |
|  | glare | 3 | 74.26 ( 23.46 - 235.01 ) | 74.23 ( 209.17 ) | 71.67 ( 27.33 ) | 6.16 ( 4.47 ) |
|  | posterior capsule opacification | 3 | 81.1 ( 25.58 - 257.14 ) | 81.07 ( 228.23 ) | 78.02 ( 29.71 ) | 6.29 ( 4.59 ) |
|  | lagophthalmos | 3 | 85.6 ( 26.97 - 271.75 ) | 85.57 ( 240.71 ) | 82.19 ( 31.26 ) | 6.36 ( 4.66 ) |
|  | eye symptom | 3 | 52.23 ( 16.6 - 164.32 ) | 52.21 ( 146.96 ) | 50.94 ( 19.52 ) | 5.67 ( 3.98 ) |
|  | erythema of eyelid | 3 | 5.04 ( 1.62 - 15.64 ) | 5.03 ( 9.67 ) | 5.02 ( 1.95 ) | 2.33 ( 0.66 ) |
|  | presbyopia | 3 | 57.6 ( 18.28 - 181.48 ) | 57.58 ( 162.24 ) | 56.04 ( 21.45 ) | 5.81 ( 4.12 ) |
|  | corneal thickening | 3 | 440.25 ( 126.49 - 1532.28 ) | 440.07 ( 1082.3 ) | 362.59 ( 127.7 ) | 8.5 ( 6.7 ) |
|  | macular hole | 2 | 15.8 ( 3.93 - 63.53 ) | 15.8 ( 27.51 ) | 15.68 ( 4.9 ) | 3.97 ( 2.3 ) |
|  | halo vision | 2 | 20.14 ( 5 - 81.09 ) | 20.13 ( 36.01 ) | 19.95 ( 6.22 ) | 4.32 ( 2.64 ) |
|  | cystoid macular oedema | 2 | 9.88 ( 2.46 - 39.63 ) | 9.87 ( 15.87 ) | 9.83 ( 3.07 ) | 3.3 ( 1.63 ) |
|  | cataract nuclear | 2 | 30.66 ( 7.59 - 123.89 ) | 30.65 ( 56.53 ) | 30.22 ( 9.39 ) | 4.92 ( 3.23 ) |
|  | corneal degeneration | 2 | 70.83 ( 17.3 - 290.08 ) | 70.82 ( 133.07 ) | 68.49 ( 21.05 ) | 6.1 ( 4.39 ) |
|  | photopsia | 2 | 3.51 ( 0.88 - 14.03 ) | 3.5 ( 3.57 ) | 3.5 ( 1.1 ) | 1.81 ( 0.14 ) |
|  | macular fibrosis | 2 | 36.36 ( 8.98 - 147.19 ) | 36.35 ( 67.56 ) | 35.73 ( 11.09 ) | 5.16 ( 3.47 ) |
|  | retinal disorder | 2 | 9.17 ( 2.29 - 36.79 ) | 9.17 ( 14.49 ) | 9.13 ( 2.86 ) | 3.19 ( 1.52 ) |
|  | vitreous detachment | 2 | 8.22 ( 2.05 - 32.95 ) | 8.21 ( 12.62 ) | 8.19 ( 2.56 ) | 3.03 ( 1.36 ) |
|  | lacrimal disorder | 2 | 34.82 ( 8.6 - 140.88 ) | 34.81 ( 64.58 ) | 34.24 ( 10.63 ) | 5.1 ( 3.41 ) |
|  | deposit eye | 2 | 85.59 ( 20.8 - 352.23 ) | 85.57 ( 160.48 ) | 82.19 ( 25.16 ) | 6.36 ( 4.65 ) |
|  | iritis | 2 | 6.12 ( 1.53 - 24.54 ) | 6.12 ( 8.54 ) | 6.11 ( 1.91 ) | 2.61 ( 0.94 ) |
|  | conjunctival oedema | 2 | 21.07 ( 5.23 - 84.86 ) | 21.06 ( 37.83 ) | 20.86 ( 6.5 ) | 4.38 ( 2.7 ) |
|  | dermatochalasis | 2 | 65.21 ( 15.95 - 266.56 ) | 65.2 ( 122.53 ) | 63.22 ( 19.46 ) | 5.98 ( 4.28 ) |
|  | conjunctival haemorrhage | 2 | 5.53 ( 1.38 - 22.15 ) | 5.53 ( 7.4 ) | 5.52 ( 1.73 ) | 2.46 ( 0.79 ) |
|  | limbal stem cell deficiency | 1 | 79 ( 10.72 - 582.24 ) | 78.99 ( 74.15 ) | 76.1 ( 14.31 ) | 6.25 ( 4.5 ) |
|  | strabismus | 1 | 3.64 ( 0.51 - 25.9 ) | 3.64 ( 1.91 ) | 3.64 ( 0.7 ) | 1.86 ( 0.19 ) |
|  | corneal pigmentation | 1 | 342.32 ( 41.21 - 2843.85 ) | 342.28 ( 291.67 ) | 293.52 ( 49.92 ) | 8.2 ( 6.24 ) |
|  | keratic precipitates | 1 | 9.55 ( 1.34 - 68.14 ) | 9.55 ( 7.62 ) | 9.51 ( 1.84 ) | 3.25 ( 1.57 ) |
|  | tractional retinal detachment | 1 | 39.5 ( 5.46 - 285.76 ) | 39.49 ( 36.81 ) | 38.77 ( 7.4 ) | 5.28 ( 3.56 ) |
|  | corneal scar | 1 | 19.02 ( 2.65 - 136.26 ) | 19.02 ( 16.91 ) | 18.85 ( 3.63 ) | 4.24 ( 2.55 ) |
|  | vitreous haemorrhage | 1 | 3.94 ( 0.55 - 28.04 ) | 3.94 ( 2.19 ) | 3.94 ( 0.76 ) | 1.98 ( 0.31 ) |
|  | anterior chamber disorder | 1 | 62.24 ( 8.51 - 455.14 ) | 62.23 ( 58.48 ) | 60.43 ( 11.44 ) | 5.92 ( 4.18 ) |
|  | pupillary disorder | 1 | 19.94 ( 2.78 - 142.94 ) | 19.94 ( 17.82 ) | 19.76 ( 3.8 ) | 4.3 ( 2.61 ) |
|  | corneal endothelial cell loss | 1 | 102.7 ( 13.78 - 765.33 ) | 102.68 ( 95.9 ) | 97.84 ( 18.22 ) | 6.61 ( 4.84 ) |
|  | corneal infiltrates | 1 | 45.64 ( 6.29 - 331.17 ) | 45.64 ( 42.71 ) | 44.67 ( 8.51 ) | 5.48 ( 3.76 ) |
|  | conjunctivochalasis | 1 | 256.74 ( 32.11 - 2053.06 ) | 256.71 ( 226.41 ) | 228.3 ( 40.09 ) | 7.83 ( 5.94 ) |
|  | open angle glaucoma | 1 | 23.88 ( 3.33 - 171.51 ) | 23.88 ( 21.67 ) | 23.62 ( 4.54 ) | 4.56 ( 2.87 ) |
|  | anisometropia | 1 | 205.39 ( 26.29 - 1604.74 ) | 205.37 ( 184.88 ) | 186.79 ( 33.44 ) | 7.55 ( 5.68 ) |
|  | retinopathy hypertensive | 1 | 37.34 ( 5.17 - 269.9 ) | 37.34 ( 34.73 ) | 36.69 ( 7.01 ) | 5.2 ( 3.49 ) |
|  | vitreous degeneration | 1 | 50.1 ( 6.89 - 364.24 ) | 50.09 ( 46.96 ) | 48.92 ( 9.3 ) | 5.61 ( 3.89 ) |
|  | retinal degeneration | 1 | 5.35 ( 0.75 - 38.07 ) | 5.35 ( 3.53 ) | 5.34 ( 1.03 ) | 2.42 ( 0.74 ) |
|  | epiretinal membrane | 1 | 18.34 ( 2.56 - 131.35 ) | 18.34 ( 16.25 ) | 18.18 ( 3.5 ) | 4.18 ( 2.5 ) |
|  | optic disc disorder | 1 | 102.7 ( 13.78 - 765.33 ) | 102.68 ( 95.9 ) | 97.84 ( 18.22 ) | 6.61 ( 4.84 ) |
|  | iris disorder | 1 | 41.08 ( 5.67 - 297.41 ) | 41.07 ( 38.33 ) | 40.29 ( 7.69 ) | 5.33 ( 3.62 ) |
|  | anterior chamber inflammation | 1 | 10.37 ( 1.45 - 74.02 ) | 10.37 ( 8.43 ) | 10.32 ( 1.99 ) | 3.37 ( 1.69 ) |
|  | anterior chamber flare | 1 | 23.34 ( 3.25 - 167.57 ) | 23.34 ( 21.14 ) | 23.09 ( 4.44 ) | 4.53 ( 2.84 ) |
|  | choroidal neovascularisation | 1 | 14.07 ( 1.97 - 100.56 ) | 14.07 ( 12.05 ) | 13.98 ( 2.7 ) | 3.81 ( 2.12 ) |
|  | corneal leukoma | 1 | 205.39 ( 26.29 - 1604.74 ) | 205.37 ( 184.88 ) | 186.79 ( 33.44 ) | 7.55 ( 5.68 ) |
|  | age-related macular degeneration | 1 | 4.5 ( 0.63 - 32.05 ) | 4.5 ( 2.72 ) | 4.5 ( 0.87 ) | 2.17 ( 0.5 ) |
|  | aphakia | 1 | 684.64 ( 71.21 - 6582.87 ) | 684.55 ( 511.92 ) | 513.66 ( 77.31 ) | 9 ( 6.9 ) |
|  | eye ulcer | 1 | 11.16 ( 1.56 - 79.68 ) | 11.16 ( 9.2 ) | 11.11 ( 2.14 ) | 3.47 ( 1.79 ) |
|  | lens disorder | 1 | 41.92 ( 5.79 - 303.6 ) | 41.91 ( 39.14 ) | 41.09 ( 7.84 ) | 5.36 ( 3.65 ) |
|  | corneal neovascularisation | 1 | 46.68 ( 6.43 - 338.86 ) | 46.67 ( 43.7 ) | 45.66 ( 8.69 ) | 5.51 ( 3.79 ) |
|  | lacrimation decreased | 1 | 23.88 ( 3.33 - 171.51 ) | 23.88 ( 21.67 ) | 23.62 ( 4.54 ) | 4.56 ( 2.87 ) |
|  | cataract diabetic | 1 | 136.93 ( 18.08 - 1036.77 ) | 136.91 ( 126.49 ) | 128.42 ( 23.6 ) | 7 ( 5.2 ) |
|  | scleritis | 1 | 4.34 ( 0.61 - 30.89 ) | 4.34 ( 2.57 ) | 4.33 ( 0.84 ) | 2.12 ( 0.44 ) |
|  | pterygium | 1 | 22.57 ( 3.14 - 161.99 ) | 22.57 ( 20.39 ) | 22.33 ( 4.29 ) | 4.48 ( 2.79 ) |
|  | pinguecula | 1 | 108.1 ( 14.47 - 807.64 ) | 108.09 ( 100.79 ) | 102.73 ( 19.09 ) | 6.68 ( 4.9 ) |
|  | vitreous opacities | 1 | 5.11 ( 0.72 - 36.36 ) | 5.11 ( 3.3 ) | 5.1 ( 0.99 ) | 2.35 ( 0.68 ) |
|  | altered visual depth perception | 1 | 14.26 ( 2 - 101.96 ) | 14.26 ( 12.25 ) | 14.17 ( 2.73 ) | 3.82 ( 2.14 ) |
|  | sudden visual loss | 1 | 30.66 ( 4.26 - 220.86 ) | 30.65 ( 28.26 ) | 30.22 ( 5.79 ) | 4.92 ( 3.22 ) |
| Brentuximab Vedotin | corneal deposits | 3 | 16.19 ( 5.2 - 50.4 ) | 16.18 ( 42.42 ) | 16.07 ( 6.21 ) | 4.01 ( 2.33 ) |
|  | refraction disorder | 1 | 11.3 ( 1.58 - 80.6 ) | 11.29 ( 9.33 ) | 11.24 ( 2.17 ) | 3.49 ( 1.81 ) |
|  | vitreous degeneration | 1 | 23.07 ( 3.22 - 165.51 ) | 23.07 ( 20.89 ) | 22.84 ( 4.39 ) | 4.51 ( 2.82 ) |
|  | purtscher retinopathy | 1 | 36.15 ( 5.01 - 260.83 ) | 36.14 ( 33.61 ) | 35.57 ( 6.81 ) | 5.15 ( 3.45 ) |
|  | ectropion | 1 | 9.95 ( 1.39 - 70.95 ) | 9.95 ( 8.01 ) | 9.91 ( 1.91 ) | 3.31 ( 1.63 ) |
|  | visual acuity reduced transiently | 1 | 9.51 ( 1.33 - 67.82 ) | 9.51 ( 7.58 ) | 9.47 ( 1.83 ) | 3.24 ( 1.57 ) |
|  | meibomianitis | 1 | 48.19 ( 6.64 - 349.65 ) | 48.19 ( 45.21 ) | 47.17 ( 8.98 ) | 5.56 ( 3.84 ) |
|  | vogt-koyanagi-harada disease | 1 | 10.9 ( 1.53 - 77.75 ) | 10.9 ( 8.94 ) | 10.85 ( 2.1 ) | 3.44 ( 1.76 ) |
|  | orbital oedema | 1 | 6.23 ( 0.88 - 44.37 ) | 6.23 ( 4.38 ) | 6.22 ( 1.2 ) | 2.64 ( 0.96 ) |
|  | blindness cortical | 1 | 7.56 ( 1.06 - 53.83 ) | 7.56 ( 5.67 ) | 7.53 ( 1.46 ) | 2.91 ( 1.24 ) |
| Enfortumab Vedotin | dry eye | 25 | 4.6 ( 3.1 - 6.81 ) | 4.58 ( 70 ) | 4.58 ( 3.3 ) | 2.19 ( 0.53 ) |
|  | vision blurred | 20 | 1.66 ( 1.07 - 2.57 ) | 1.66 ( 5.21 ) | 1.66 ( 1.15 ) | 0.73 ( -0.94 ) |
|  | lacrimation increased | 13 | 4.1 ( 2.38 - 7.07 ) | 4.1 ( 30.4 ) | 4.09 ( 2.59 ) | 2.03 ( 0.37 ) |
|  | eye discharge | 5 | 4.71 ( 1.96 - 11.32 ) | 4.7 ( 14.56 ) | 4.7 ( 2.25 ) | 2.23 ( 0.56 ) |
|  | ocular toxicity | 4 | 35.26 ( 13.15 - 94.54 ) | 35.24 ( 131.4 ) | 34.81 ( 15.25 ) | 5.12 ( 3.45 ) |
|  | periorbital oedema | 3 | 6.87 ( 2.21 - 21.33 ) | 6.87 ( 15 ) | 6.85 ( 2.65 ) | 2.78 ( 1.11 ) |
|  | blepharitis | 2 | 4.75 ( 1.19 - 19.03 ) | 4.75 ( 5.91 ) | 4.75 ( 1.49 ) | 2.25 ( 0.58 ) |
|  | keratitis | 2 | 6.82 ( 1.7 - 27.31 ) | 6.82 ( 9.9 ) | 6.8 ( 2.13 ) | 2.77 ( 1.1 ) |
|  | conjunctival hyperaemia | 2 | 5.77 ( 1.44 - 23.09 ) | 5.76 ( 7.86 ) | 5.75 ( 1.8 ) | 2.52 ( 0.86 ) |
|  | abnormal sensation in eye | 2 | 7.2 ( 1.8 - 28.86 ) | 7.2 ( 10.65 ) | 7.19 ( 2.25 ) | 2.85 ( 1.18 ) |
|  | night blindness | 1 | 4.68 ( 0.66 - 33.26 ) | 4.68 ( 2.89 ) | 4.67 ( 0.9 ) | 2.22 ( 0.55 ) |
|  | retinopathy hypertensive | 1 | 38.38 ( 5.33 - 276.17 ) | 38.37 ( 35.91 ) | 37.87 ( 7.26 ) | 5.24 ( 3.54 ) |
|  | noninfective conjunctivitis | 1 | 59.61 ( 8.22 - 432.12 ) | 59.6 ( 56.42 ) | 58.38 ( 11.13 ) | 5.87 ( 4.15 ) |
|  | eye symptom | 1 | 18.8 ( 2.63 - 134.39 ) | 18.8 ( 16.74 ) | 18.68 ( 3.6 ) | 4.22 ( 2.54 ) |
|  | eyelid disorder | 1 | 4.76 ( 0.67 - 33.89 ) | 4.76 ( 2.97 ) | 4.76 ( 0.92 ) | 2.25 ( 0.58 ) |
|  | corneal disorder | 1 | 4.96 ( 0.7 - 35.27 ) | 4.96 ( 3.15 ) | 4.95 ( 0.96 ) | 2.31 ( 0.64 ) |
| Mirvetuximab Soravtansine | vision blurred | 21 | 26.61 ( 17.15 - 41.3 ) | 25.33 ( 490.67 ) | 25.28 ( 17.5 ) | 4.66 ( 2.99 ) |
|  | keratitis | 8 | 378.61 ( 186.14 - 770.06 ) | 371.38 ( 2868.6 ) | 360.52 ( 199.04 ) | 8.49 ( 6.81 ) |
|  | dry eye | 7 | 17.06 ( 8.08 - 36.03 ) | 16.79 ( 103.91 ) | 16.77 ( 8.97 ) | 4.07 ( 2.4 ) |
|  | cataract | 4 | 9.53 ( 3.56 - 25.51 ) | 9.44 ( 30.21 ) | 9.44 ( 4.14 ) | 3.24 ( 1.57 ) |
|  | eye pain | 4 | 10.52 ( 3.93 - 28.16 ) | 10.43 ( 34.09 ) | 10.42 ( 4.57 ) | 3.38 ( 1.71 ) |
|  | eye irritation | 4 | 11.05 ( 4.13 - 29.6 ) | 10.96 ( 36.19 ) | 10.95 ( 4.8 ) | 3.45 ( 1.78 ) |
|  | photophobia | 3 | 24.1 ( 7.73 - 75.11 ) | 23.93 ( 65.82 ) | 23.89 ( 9.23 ) | 4.58 ( 2.9 ) |
|  | eye disorder | 3 | 14.21 ( 4.56 - 44.28 ) | 14.12 ( 36.54 ) | 14.1 ( 5.45 ) | 3.82 ( 2.15 ) |
|  | corneal deposits | 2 | 618.05 ( 148.88 - 2565.65 ) | 615.1 ( 1167.81 ) | 585.85 ( 178.05 ) | 9.19 ( 7.47 ) |
|  | punctate keratitis | 2 | 224.74 ( 55.32 - 912.99 ) | 223.67 ( 435.44 ) | 219.69 ( 67.99 ) | 7.78 ( 6.09 ) |
|  | uveitis | 2 | 13.78 ( 3.43 - 55.31 ) | 13.71 ( 23.56 ) | 13.7 ( 4.28 ) | 3.78 ( 2.1 ) |
|  | ocular toxicity | 2 | 240.02 ( 59.03 - 975.86 ) | 238.87 ( 464.74 ) | 234.34 ( 72.47 ) | 7.87 ( 6.18 ) |
|  | blepharitis | 1 | 34.64 ( 4.85 - 247.15 ) | 34.56 ( 32.5 ) | 34.46 ( 6.66 ) | 5.11 ( 3.43 ) |
|  | blindness | 1 | 3.73 ( 0.52 - 26.52 ) | 3.72 ( 1.99 ) | 3.72 ( 0.72 ) | 1.89 ( 0.22 ) |
|  | keratopathy | 1 | 36.37 ( 5.1 - 259.58 ) | 36.29 ( 34.22 ) | 36.19 ( 6.99 ) | 5.18 ( 3.5 ) |
|  | lacrimation increased | 1 | 4.45 ( 0.62 - 31.66 ) | 4.44 ( 2.67 ) | 4.44 ( 0.86 ) | 2.15 ( 0.48 ) |
|  | corneal irritation | 1 | 1233.14 ( 157.5 - 9654.92 ) | 1230.19 ( 1116.54 ) | 1118.45 ( 199.89 ) | 10.13 ( 8.26 ) |
|  | visual acuity reduced | 1 | 11.92 ( 1.67 - 84.93 ) | 11.9 ( 9.97 ) | 11.89 ( 2.3 ) | 3.57 ( 1.9 ) |
|  | retinal haemorrhage | 1 | 31.94 ( 4.48 - 227.89 ) | 31.87 ( 29.83 ) | 31.79 ( 6.14 ) | 4.99 ( 3.31 ) |
|  | pseudophakia | 1 | 3082.85 ( 343.83 - 27641.05 ) | 3075.48 ( 2458.78 ) | 2460.58 ( 392.61 ) | 11.26 ( 9.22 ) |
|  | corneal epithelial microcysts | 1 | 397.78 ( 54.18 - 2920.72 ) | 396.84 ( 382.5 ) | 384.47 ( 72.51 ) | 8.59 ( 6.84 ) |
| Sacituzumab Govitecan | periorbital oedema | 4 | 7.13 ( 2.67 - 19.05 ) | 7.13 ( 20.96 ) | 7.1 ( 3.12 ) | 2.83 ( 1.16 ) |
|  | eyelid disorder | 1 | 3.38 ( 0.47 - 24.03 ) | 3.38 ( 1.67 ) | 3.37 ( 0.65 ) | 1.75 ( 0.08 ) |
|  | optic nerve disorder | 1 | 6.51 ( 0.91 - 46.41 ) | 6.51 ( 4.64 ) | 6.48 ( 1.25 ) | 2.7 ( 1.02 ) |
| Trastuzumab Deruxtecan | keratitis | 5 | 13.15 ( 5.44 - 31.82 ) | 13.15 ( 55.29 ) | 12.97 ( 6.19 ) | 3.7 ( 2.02 ) |
|  | visual acuity reduced | 4 | 2.76 ( 1.03 - 7.37 ) | 2.76 ( 4.47 ) | 2.75 ( 1.21 ) | 1.46 ( -0.21 ) |
|  | eye haematoma | 3 | 78.67 ( 24.16 - 256.21 ) | 78.64 ( 211.32 ) | 72.35 ( 26.94 ) | 6.18 ( 4.45 ) |
|  | ocular toxicity | 3 | 19.38 ( 6.17 - 60.85 ) | 19.38 ( 51.17 ) | 18.98 ( 7.29 ) | 4.25 ( 2.56 ) |
|  | retinal exudates | 2 | 48.19 ( 11.61 - 199.98 ) | 48.18 ( 87.66 ) | 45.76 ( 13.91 ) | 5.52 ( 3.79 ) |
|  | corneal disorder | 1 | 4.64 ( 0.65 - 33.13 ) | 4.64 ( 2.84 ) | 4.62 ( 0.89 ) | 2.21 ( 0.53 ) |
|  | excessive eye blinking | 1 | 6.86 ( 0.96 - 49.05 ) | 6.86 ( 4.96 ) | 6.81 ( 1.31 ) | 2.77 ( 1.08 ) |
|  | corneal scar | 1 | 17.83 ( 2.46 - 129.07 ) | 17.83 ( 15.57 ) | 17.5 ( 3.34 ) | 4.13 ( 2.42 ) |
|  | punctate keratitis | 1 | 6.96 ( 0.97 - 49.82 ) | 6.96 ( 5.07 ) | 6.92 ( 1.33 ) | 2.79 ( 1.1 ) |
| Trastuzumab Emtansine | vision blurred | 42 | 1.67 ( 1.24 - 2.27 ) | 1.67 ( 11.37 ) | 1.67 ( 1.3 ) | 0.74 ( -0.92 ) |
|  | dry eye | 24 | 2.79 ( 1.87 - 4.17 ) | 2.79 ( 27.53 ) | 2.79 ( 1.99 ) | 1.48 ( -0.19 ) |
|  | lacrimation increased | 17 | 2.95 ( 1.84 - 4.75 ) | 2.95 ( 21.93 ) | 2.95 ( 1.98 ) | 1.56 ( -0.11 ) |
|  | corneal disorder | 8 | 20.82 ( 10.39 - 41.74 ) | 20.81 ( 149.94 ) | 20.69 ( 11.56 ) | 4.37 ( 2.7 ) |
|  | retinal detachment | 6 | 3.58 ( 1.61 - 7.97 ) | 3.58 ( 11.13 ) | 3.57 ( 1.83 ) | 1.84 ( 0.17 ) |
|  | blindness unilateral | 6 | 2.39 ( 1.07 - 5.32 ) | 2.39 ( 4.83 ) | 2.39 ( 1.22 ) | 1.25 ( -0.41 ) |
|  | asthenopia | 4 | 3.73 ( 1.4 - 9.94 ) | 3.73 ( 7.97 ) | 3.72 ( 1.64 ) | 1.9 ( 0.23 ) |
|  | astigmatism | 4 | 13.49 ( 5.05 - 36.02 ) | 13.49 ( 46.06 ) | 13.44 ( 5.91 ) | 3.75 ( 2.08 ) |
|  | periorbital oedema | 4 | 4.32 ( 1.62 - 11.53 ) | 4.32 ( 10.2 ) | 4.32 ( 1.9 ) | 2.11 ( 0.44 ) |
|  | hypermetropia | 3 | 16.28 ( 5.24 - 50.63 ) | 16.28 ( 42.81 ) | 16.2 ( 6.27 ) | 4.02 ( 2.35 ) |
|  | conjunctival hyperaemia | 3 | 4.63 ( 1.49 - 14.37 ) | 4.63 ( 8.52 ) | 4.62 ( 1.79 ) | 2.21 ( 0.54 ) |
|  | corneal deposits | 3 | 27.52 ( 8.83 - 85.75 ) | 27.52 ( 76.04 ) | 27.3 ( 10.55 ) | 4.77 ( 3.1 ) |
|  | myopia | 3 | 6.86 ( 2.21 - 21.29 ) | 6.86 ( 14.97 ) | 6.84 ( 2.65 ) | 2.77 ( 1.11 ) |
|  | keratitis | 2 | 3.75 ( 0.94 - 15.02 ) | 3.75 ( 4.04 ) | 3.75 ( 1.18 ) | 1.91 ( 0.24 ) |
|  | scintillating scotoma | 2 | 49.24 ( 12.19 - 198.93 ) | 49.23 ( 93.14 ) | 48.53 ( 15.09 ) | 5.6 ( 3.92 ) |
|  | corneal erosion | 2 | 24.71 ( 6.15 - 99.33 ) | 24.71 ( 45.16 ) | 24.53 ( 7.66 ) | 4.62 ( 2.94 ) |
|  | corneal oedema | 2 | 4.63 ( 1.16 - 18.55 ) | 4.63 ( 5.69 ) | 4.63 ( 1.45 ) | 2.21 ( 0.54 ) |
|  | ocular vascular disorder | 2 | 14.85 ( 3.7 - 59.56 ) | 14.85 ( 25.71 ) | 14.79 ( 4.62 ) | 3.89 ( 2.21 ) |
|  | ulcerative keratitis | 2 | 4.01 ( 1 - 16.06 ) | 4.01 ( 4.52 ) | 4.01 ( 1.26 ) | 2 ( 0.34 ) |
|  | optic neuropathy | 2 | 5.62 ( 1.4 - 22.51 ) | 5.62 ( 7.59 ) | 5.61 ( 1.76 ) | 2.49 ( 0.82 ) |
|  | dacryostenosis acquired | 2 | 7.79 ( 1.94 - 31.19 ) | 7.79 ( 11.8 ) | 7.77 ( 2.43 ) | 2.96 ( 1.29 ) |
|  | punctate keratitis | 2 | 11.81 ( 2.95 - 47.35 ) | 11.81 ( 19.72 ) | 11.77 ( 3.68 ) | 3.56 ( 1.89 ) |
|  | vitreous haemorrhage | 2 | 3.99 ( 1 - 15.97 ) | 3.99 ( 4.48 ) | 3.99 ( 1.25 ) | 2 ( 0.33 ) |
|  | corneal exfoliation | 1 | 62 ( 8.58 - 448.24 ) | 62 ( 58.92 ) | 60.89 ( 11.63 ) | 5.93 ( 4.22 ) |
|  | lacrimation decreased | 1 | 11.01 ( 1.55 - 78.45 ) | 11.01 ( 9.07 ) | 10.98 ( 2.12 ) | 3.46 ( 1.78 ) |
|  | corneal opacity | 1 | 4.16 ( 0.59 - 29.6 ) | 4.16 ( 2.4 ) | 4.16 ( 0.81 ) | 2.06 ( 0.39 ) |
|  | orbital oedema | 1 | 10.7 ( 1.5 - 76.19 ) | 10.7 ( 8.76 ) | 10.67 ( 2.06 ) | 3.41 ( 1.74 ) |
|  | lid sulcus deepened | 1 | 11.35 ( 1.59 - 80.85 ) | 11.35 ( 9.41 ) | 11.31 ( 2.19 ) | 3.5 ( 1.83 ) |
|  | retinal vascular disorder | 1 | 10.43 ( 1.46 - 74.28 ) | 10.43 ( 8.5 ) | 10.4 ( 2.01 ) | 3.38 ( 1.7 ) |
|  | corneal irritation | 1 | 55.8 ( 7.73 - 402.69 ) | 55.8 ( 52.93 ) | 54.9 ( 10.5 ) | 5.78 ( 4.07 ) |
|  | refraction disorder | 1 | 19.13 ( 2.68 - 136.6 ) | 19.13 ( 17.09 ) | 19.03 ( 3.67 ) | 4.25 ( 2.57 ) |
|  | amblyopia | 1 | 10.4 ( 1.46 - 74.05 ) | 10.4 ( 8.47 ) | 10.37 ( 2.01 ) | 3.37 ( 1.7 ) |
|  | chalazion | 1 | 8.59 ( 1.21 - 61.11 ) | 8.58 ( 6.68 ) | 8.56 ( 1.66 ) | 3.1 ( 1.43 ) |
|  | meibomian gland dysfunction | 1 | 12.04 ( 1.69 - 85.81 ) | 12.04 ( 10.09 ) | 12 ( 2.32 ) | 3.59 ( 1.91 ) |
|  | vogt-koyanagi-harada disease | 1 | 17.81 ( 2.5 - 127.11 ) | 17.81 ( 15.78 ) | 17.72 ( 3.42 ) | 4.15 ( 2.47 ) |
|  | corneal toxicity | 1 | 58.74 ( 8.13 - 424.25 ) | 58.74 ( 55.77 ) | 57.74 ( 11.04 ) | 5.85 ( 4.14 ) |
|  | retinal exudates | 1 | 6.76 ( 0.95 - 48.12 ) | 6.76 ( 4.9 ) | 6.75 ( 1.31 ) | 2.76 ( 1.08 ) |
|  | corneal epithelial wrinkling | 1 | 418.52 ( 52.34 - 3346.61 ) | 418.49 ( 370.21 ) | 372.1 ( 65.34 ) | 8.54 ( 6.64 ) |
|  | retinal vein thrombosis | 1 | 10.53 ( 1.48 - 74.98 ) | 10.53 ( 8.6 ) | 10.5 ( 2.03 ) | 3.39 ( 1.72 ) |
|  | corneal cyst | 1 | 56.75 ( 7.86 - 409.63 ) | 56.74 ( 53.85 ) | 55.82 ( 10.68 ) | 5.8 ( 4.1 ) |
|  | anisocoria | 1 | 4 ( 0.56 - 28.4 ) | 4 ( 2.24 ) | 3.99 ( 0.77 ) | 2 ( 0.33 ) |
|  | eye ulcer | 1 | 7.54 ( 1.06 - 53.66 ) | 7.54 ( 5.66 ) | 7.53 ( 1.46 ) | 2.91 ( 1.24 ) |
